# New Zealand glowworm (*Arachnocampa luminosa*) bioluminescence is produced by a firefly-like luciferase but an entirely new luciferin

**DOI:** 10.1038/s41598-018-21298-w

**Published:** 2018-02-19

**Authors:** Oliver C. Watkins, Miriam L. Sharpe, Nigel B. Perry, Kurt L. Krause

**Affiliations:** 1 Department of Biochemistry, University of Otago, Dunedin, New Zealand; 2 New Zealand Institute for Plant and Food Research Ltd., Department of Chemistry, University of Otago, Dunedin, New Zealand

## Abstract

The New Zealand glowworm, *Arachnocampa luminosa*, is well-known for displays of blue-green bioluminescence, but details of its bioluminescent chemistry have been elusive. The glowworm is evolutionarily distant from other bioluminescent creatures studied in detail, including the firefly. We have isolated and characterised the molecular components of the glowworm luciferase-luciferin system using chromatography, mass spectrometry and ^1^H NMR spectroscopy. The purified luciferase enzyme is in the same protein family as firefly luciferase (31% sequence identity). However, the luciferin substrate of this enzyme is produced from xanthurenic acid and tyrosine, and is entirely different to that of the firefly and known luciferins of other glowing creatures. A candidate luciferin structure is proposed, which needs to be confirmed by chemical synthesis and bioluminescence assays. These findings show that luciferases can evolve independently from the same family of enzymes to produce light using structurally different luciferins.

## Introduction

Glowworms are found in New Zealand and Australia, and are a major tourist attraction at sites located across both countries. In contrast to luminescent beetles such as the firefly (Coleoptera), whose bioluminescence has been well characterised (reviewed by ref.^1^), the molecular details of glowworm bioluminescence have remained elusive. These glowworms are the larvae of fungus gnats of the genus *Arachnocampa*, with eight species endemic to Australia and a single species found only in New Zealand^2^. The New Zealand *Arachnocampa luminosa* (Skuse) (Diptera: Keroplatidae) is found in damp areas of forest, by streams and in caves throughout the country (the Māori name *titiwai* means “projected over water”). The larvae attract prey using blue-green light emitted from a specialised light organ found at the posterior end of the body, derived from modified Malpighian tubules^3,4^.

Bioluminescent systems create light through the oxidation of a small molecule substrate, generically known as a luciferin, catalysing the reaction using an enzyme called a luciferase^5,6^. The reaction produces a high-energy singlet state intermediate, which is able to release energy as visible light, or pass it onto a secondary emitter^6,7^. There are over 40 different bioluminescent systems^1,8^, with a great variety in the structure, mechanism and substrate specificity of the luciferase enzymes (reviewed by ref.^9^) and also in the structures of the luciferins. Nine different luciferin structures have been solved^10,11^, most built around four natural product classes: aromatic amino acids, flavins, tetrapyrroles and quinones.

The fireflies mostly produce yellow-green light (λ_max_ 552–582 nm) through the oxidative adenylation of firefly D-luciferin^1^. This reaction is catalysed by firefly luciferase, a member of the ANL superfamily of adenylating enzymes (**A**cyl-CoA synthetases, **N**onribosomal peptide synthetase adenylation domains, beetle **L**uciferases). ANL enzymes catalyse a two-step reaction^12^; initially activating a carboxylate substrate with ATP (adenosine triphosphate) to form an adenylate intermediate, followed by the formation of a thioester by the NRPS and acyl-CoA synthetases. In the reaction catalysed by firefly luciferase, the second partial reaction is the oxidative decarboxylation of the activated luciferyl-adenylate, producing a high-energy dioxetanone which decomposes to release carbon dioxide and a photon^11^.

In *Arachnocampa*, studies by Lee (working with *A. richardsae*), Shimomura (*A. luminosa*) and Viviani *et al*. (*A. flava*) have established that the bioluminescence is also a luciferin-luciferase reaction that requires ATP-Mg^2+^ and oxygen^13–15^. Silva *et al*.^16^ carried out a transcriptional profiling study on *A. luminosa*, sequencing 537 cDNAs that were constructed from light organ expressed sequence tags using the Sanger method (they did not include a comparison between tissues). Our research on *A. luminosa* using transcriptome sequencing and differential analysis of expression from the light organ compared with non-light organ tissue, revealed three candidate glowworm luciferases, all from the same superfamily of enzymes as the firefly luciferase^17^.

A recent study of bioluminescence in Australian glowworms reported the identification of the *A. richardsae* luciferase^18^. The authors isolated a sequence from a cDNA library made from light organ RNA that encoded an enzyme from the ANL superfamily. The study also reported the presence of firefly D-luciferin in the glow-worm light organs, and further, that the cloned enzyme expressed recombinantly in bacteria was able to produce light using firefly D-luciferin, which is in contrast to previous studies reporting no cross reaction between the glowworm and firefly bioluminescent systems^13,15^.

The glowworm and firefly bioluminescent systems have evolutionarily distinct origins: glowworms are true flies, of the order Diptera (family Keroplatidae), whereas the well-studied fireflies are beetles, members of the order Coleoptera (superfamily Elateroidea). Diptera and Coleoptera diverged about 330 million years ago, and do not have any known bioluminescent species intervening^8,19,20^.

In this study we aimed to isolate and identify luciferin and luciferase from *A. luminosa* light organ tissue using biochemical techniques, including chromatographic fractionation of cell-free lysates, followed by assays and mass spectrometry (MS). We confirm that the New Zealand glowworm luciferase is a member of the ANL superfamily, similar to firefly luciferase, but reveal the *A. luminosa* luciferin to be a novel luciferin, containing tyrosine (Tyr) and xanthurenic acid (XA) moieties, and propose a candidate *A. luminosa* luciferin structure.

## Results

### Assay development and optimisation

**Figure 1:**
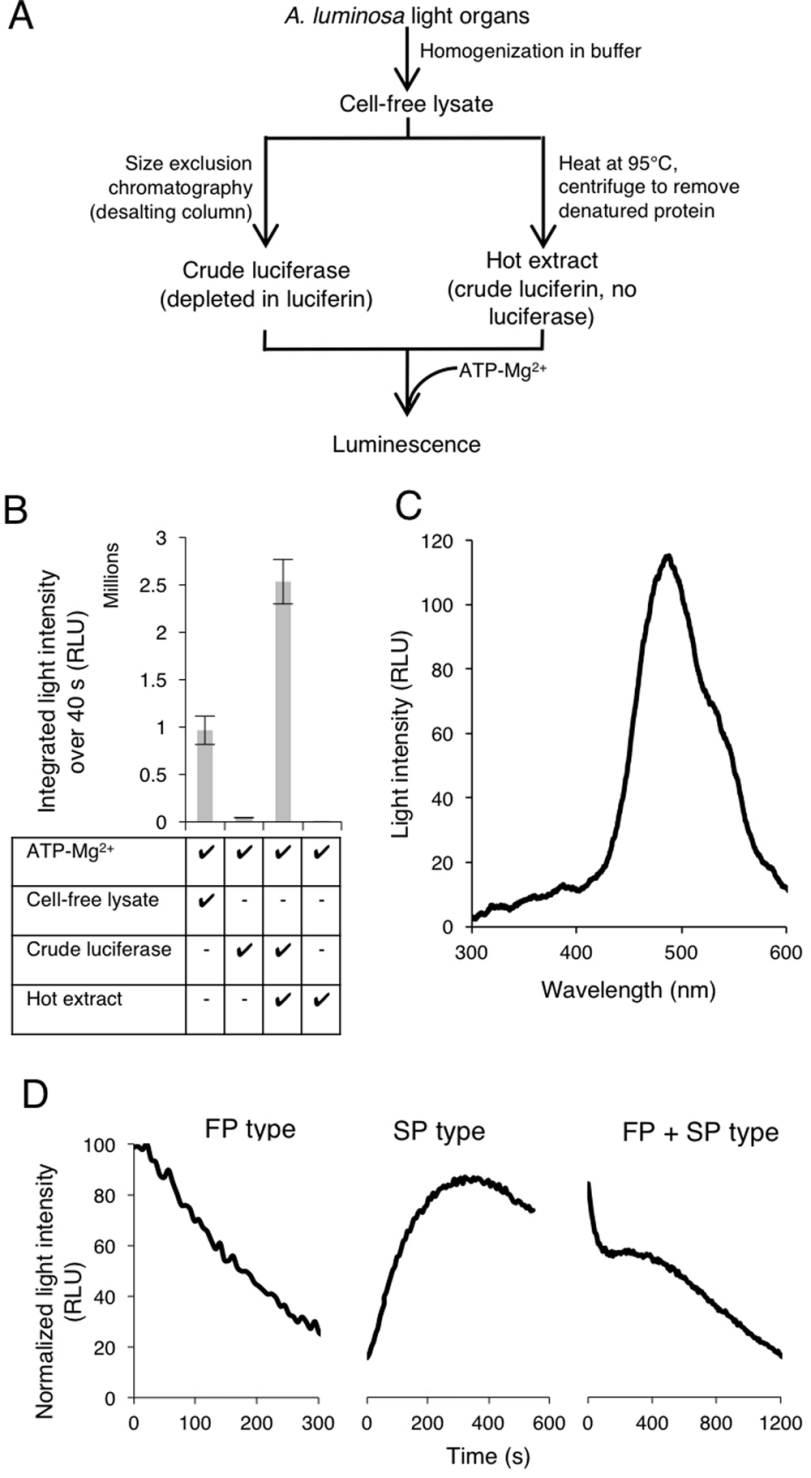
Preparation of components of luciferase and luciferin detection assays. (**A**) Overview of the production of *A. luminosa* lysate, crude luciferase and hot extract. (**B**) Luminescence of lysate, crude luciferase and hot extract for 40 s immediately after the addition of ATP-Mg^2+^. Data points represent the mean of three experiments; error bars indicate standard deviation. (**C**) *In vitro* bioluminescence spectrum of hot extract mixed with crude luciferase and ATP-Mg^2+^, with a peak emission wavelength of 487 nm. (**D**) Variations in the time course of luminescence reactions initiated by the addition of ATP-Mg^2+^ to hot extract and crude luciferase (added 5 s before measurements started). From left: ‘fast peak’ (FP) type, ‘slow peak’ (SP) type, and intermediate FP + SP type. RLU: relative luminescence units.

We developed optimised assay methods for the investigation of the *Arachnocampa* bioluminescent system based on the ‘hot extract’, ‘cold extract’ assays of *Arachnocampa* luciferin-luciferase reported by^15^. We prepared hot extract containing luciferin without luciferase by heating a cell-free cold extract lysate at 95 °C for 5 min, thereby denaturing all proteins present, but leaving heat stable small molecules intact. Crude luciferase samples depleted in luciferin and related metabolites were prepared from the cell-free cold extract lysate using a centrifugation-based desalting size exclusion column, which separates large protein molecules from small molecules. An outline of our extract preparation is provided in Fig. 1A.

Combining hot extract and crude luciferase samples with ATP-Mg^2+^ produced bioluminescence (Fig. 1B). The luminescence emission spectrum produced by this reaction (Fig. 1C) had the same peak emission wavelength (487 nm) as that reported previously for *A. luminosa*^14^. Notably, the hot extract alone produced no light when mixed with ATP-Mg^2+^, showing it to be completely depleted in active luciferase. A small amount of light was produced by the combination of crude luciferase and ATP-Mg^2+^, so these samples were not entirely depleted in luciferin. However, significantly more luminescence (>100 times) was produced with the addition of hot extract to this combination, indicating that the luciferin content was low enough to use the crude luciferase preparations in a luciferin detection assay. As a control in all luciferin assays, the luminescence produced by the sample was compared with that produced by crude luciferase and ATP-Mg^2+^ without sample addition.

Other improvements to the assay involved additives, buffer optimisation, and careful light organ preparation. The stability of lysate was improved by the addition of reducing agents such as dithiothreitol (DTT), extending the time that light was produced by at least 5-fold (Supplementary Fig. S1), and luminescence intensity was increased over 8-fold using MOPS (3-(N-morpholino)propanesulfonic acid), pH 7.5 instead of Tris (Tris(hydroxymethyl)amino methane) pH 8.0 as the assay buffer (Supplementary Fig. S2). Care was taken to include only light organs in the lysate preparation, because the addition of non-luminescent glowworm tissue other than light organ significantly reduced lysate luminescence (Supplementary Fig. S3). Also, lysate was always kept at 4 °C when not stored at −80 °C. Even with these improvements, bioluminescence from freshly produced (or thawed) light organ lysate upon the addition of hot extract and ATP-Mg^2+^ declined over several hours until it was undetectable 4 to 6 hours after lysis (or thawing).

### Time course of bioluminescence

We found different time courses of light emission from the reactions of different *A. luminosa* lysates (Fig. 1D). Some preparations produced light with a time course profile similar to those observed previously^13,15^, where light intensity increased rapidly to a maximum within seconds of adding ATP-Mg^2+^, then decreased gradually over several minutes (‘fast peak’ or FP type). However, other lysates produced profiles in which the luminescent maximum was reached many minutes after ATP-Mg^2+^ was added (‘slow peak’ or SP type). Some lysates produced profiles that were intermediate between FP and SP luminescence. Initially it was unclear what factors during lysate preparation were associated with the different luminescence time course profiles, but below we report data suggesting that these different profiles may relate to the biosynthesis of luciferin from its precursors.

### *A. luminosa* luciferase and luciferin do not cross react with firefly luciferase and luciferin

**Figure 2:**
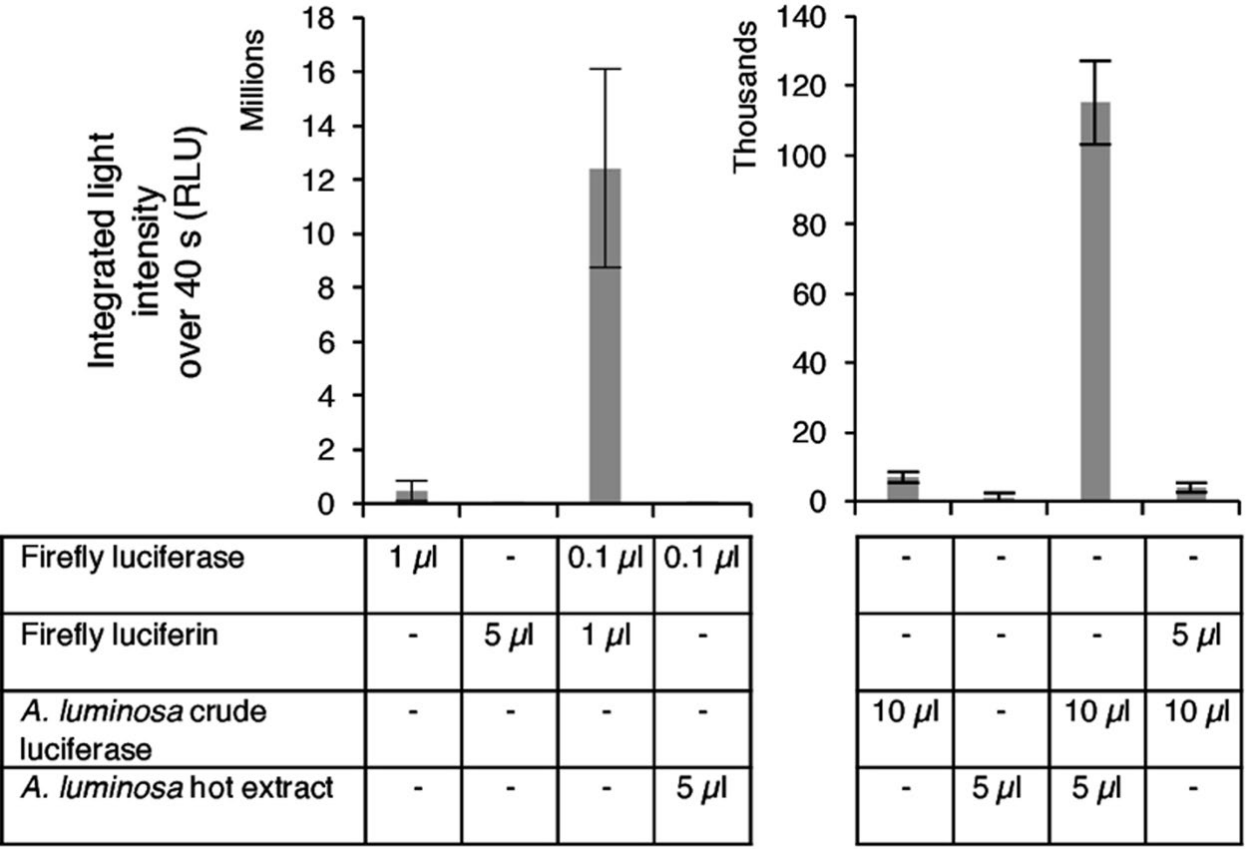
*A. luminosa* luciferase does not produce bioluminescence with firefly luciferin and vice versa. ATP-Mg^2+^ was added to all assays and luminescence measured for 40 s. Data points represent the mean of three experiments; error bars indicate standard deviation. RLU: relative luminescence units.

*A. luminosa* crude luciferase preparations were tested to see if they would produce light following the addition of firefly luciferin and ATP-Mg^2+^. Additionally, firefly luciferase was tested to see if it would produce light when mixed with *A. luminosa* hot extract and ATP-Mg^2+^. When the luciferins and luciferases from glowworm and firefly species were mixed, we detected the same level of light as for the luciferases without luciferins added (Fig. 2). This is in contrast to the intense luminescence obtained when crude *A. luminosa* luciferase and *A. luminosa* hot extract preparations were mixed together, or when firefly luciferase and firefly luciferin were mixed. Therefore, firefly luciferin is not the substrate of the *A. luminosa* bioluminescent reaction. Furthermore, analysis of *A. luminosa* lysates using MS and liquid chromatography-MS (LC-MS) detected no firefly D-luciferin ([M-H]^−^ C_11_H_7_N_2_O_3_S_2_^−^ (278.9895) or decarboxylated daughter ion C_10_H_7_N_2_OS_2_^−^ (234.9996)^21^ (Supplementary Fig. S4 and Table S1).

### Partial purification and identification of the glowworm luciferase enzyme

Protein-associated bioluminescent activity was partially purified from light organ lysate using fast protein liquid chromatography (FPLC). We used two columns sequentially during purification: a gel filtration column followed by an ion exchange column. Fractions eluted from both columns were assayed for bioluminescent activity using hot extract as luciferin substrate and analysed using SDS-PAGE.

**Figure 3:**
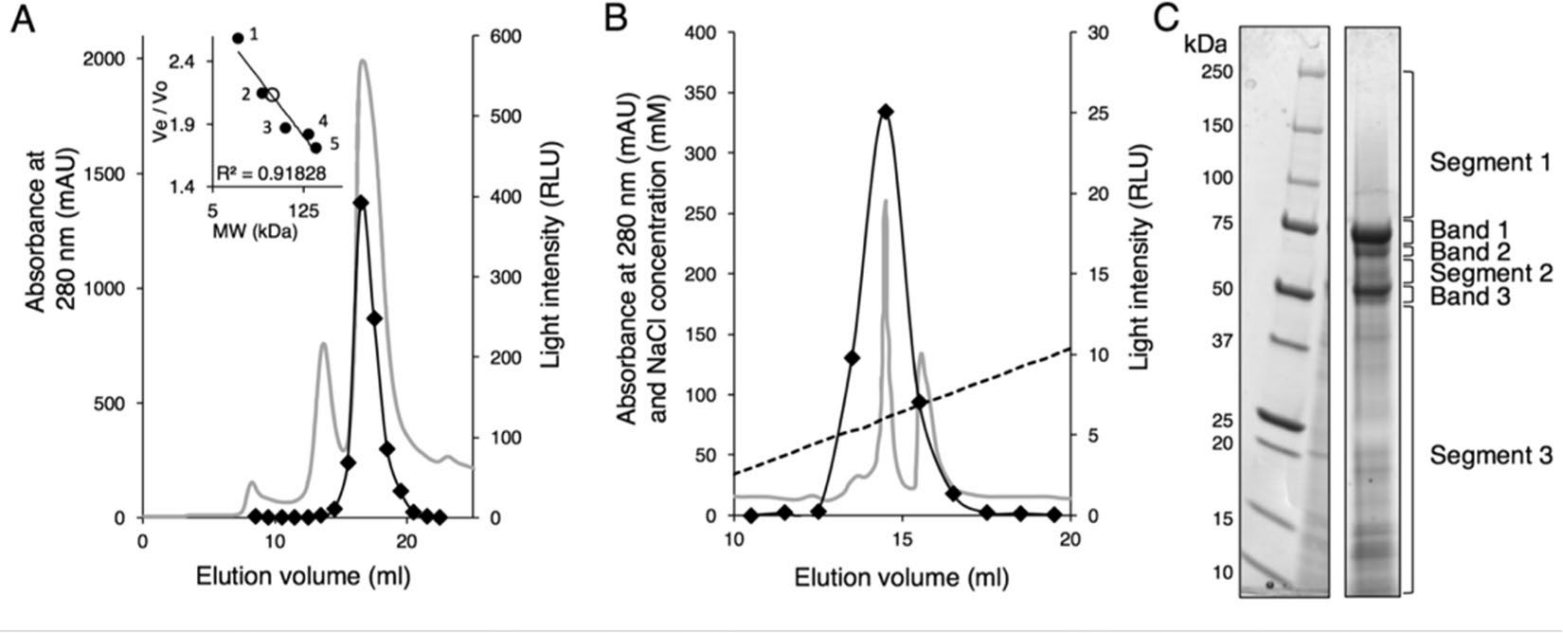
Purification of luciferase bioluminescent activity from *A. luminosa* light organ cell lysate. Absorbance at 280 nm is represented as a grey line and luminescence as a black solid line. Light intensity was the integrated relative light units recorded over the first 40 seconds of the activity assay with *A. luminosa* hot extract and ATP-Mg^2+^. (**A**) Gel filtration chromatography. See also: SDS-PAGE Gel G1 available in the supplement, Inset: calibration curve for the determination of size associated with glowworm luciferase activity, constructed using five standard proteins of known molecular weights (closed circles; 1 cytochrome C, 2 carbonic anhydrase, 3 bovine albumin, 4 alcohol dehydrogenase, 5 β-amylase). The Ve/Vo value of the fraction containing the highest luminescent activity is represented by an open circle. (**B**) Ion exchange chromatography on active fractions pooled from gel filtration chromatography, (gradient of NaCl represented as a dashed black line). (**C**) SDS-PAGE of the fraction eluted from the ion exchange column with the highest bioluminescent activity (right lane), indicating the different areas of the gel used for MS analysis. Left lane: molecular weight markers. Lanes are cropped from different parts of the same gel. See also: full SDS-PAGE gel G2 available in the supplementary information.

Five of the fractions eluting from the gel filtration column contained significant bioluminescent activity (Fig. 3A), with the highest level of activity eluting between 16 and 17 ml, correlating to a size of about 41 kDa. This peak was quite broad (size range of 11 to 63 kDa), and therefore the estimate of the molecular weight of the corresponding enzyme guided by calibration standards is very approximate. The five most active fractions were pooled and injected onto the ion exchange column.

Proteins binding to the ion exchange column were eluted with a shallow gradient, from 0 to 200 mM NaCl (Fig. 3B). Four fractions eluting at about 60 to 90 mM NaCl had detectable bioluminescent activity, and the fraction with the highest activity eluted early in the gradient. SDS-PAGE of the active fractions eluted from ion exchange chromatography showed that they contained a mixture of different proteins, including three prominent bands and many faint bands. The gel of the most active fraction is pictured in Fig. 3C. Further purification was unable to be carried out because of loss of sample during chromatography steps, and decline of the bioluminescent signal.

To identify the proteins in the most active fraction using MS, we divided the gel lane in Fig. 3C into six distinct areas: three prominent bands, and three segments that encompassed the remaining faint bands in the lane. Bands and segments of interest were excised from the gel and digested using trypsin. The masses of resultant peptides were measured using MS (MALDI TOF/TOF) and then searched against a glowworm transcriptome assembly (Genbank accession GDQV00000000)^17^ that was translated into all possible reading frames. Details of the MS analysis, including protein sequence and peptide coverage are provided in Supplementary Tables S2 and S3. Annotated sequence homologs of the glowworm proteins were found using BLASTX^22^ and the Genbank protein sequence database (NCBI; http://www.ncbi.nlm.nih.gov).

Two types of protein dominate the list of identified proteins in the active fraction: proteins annotated as larval serum protein 2 (encoded by four transcripts) and proteins annotated as luciferin 4-monooxygenase (encoded by two transcripts). Annotation as luciferin 4-monooxygenase originates from sequence similarity to firefly luciferases. Alignments of the four larval serum protein 2 sequences (Supplementary Fig. S5) show that they are four similar but separate proteins; only one of these has a transcript that provides the sequence for the full-length protein. Only one of the two luciferin 4-monooxygenase transcripts encodes a full-length protein (62762); the other (55693) appears to be a fragment. Where the two sequences can be aligned, they are nearly identical except for two amino acids (Leu29 and Ile51 in 55693, Ile29 and Val51 in 62762; Supplementary Fig. S6). It is likely that they are separate isoforms, since both amino acid variations are captured by the peptide coverage of these proteins on the gel (Supplementary Table S3). Two aldehyde dehydrogenases also recur on the list; these also appear to be isoforms, with 76% sequence identity.

Alignment of the *A. luminosa* luciferase with the *A. richardsae* luciferase sequence isolated by Trowell *et al.*^18^ shows that the sequences are very similar, with 86% sequence identity (Supplementary Fig. S7). In contrast, the *A. luminosa* luciferase encoded by transcript 62762 has 30% identity with luciferases from the fireflies *Photinus pyralis* and *Luciola cruciata*. The ATP-binding motifs conserved throughout the ANL superfamily^12^ are present in all four sequences, and the lysine residue that plays a key role in the firefly luciferase adenylation half reaction^23^ is conserved in the *Arachnocampa* sequences. However, the luciferin-binding residues from the beetle luciferase^24,25^ are not as well conserved in the *Arachnocampa* luciferase, and the lysine that plays a key role in the oxidation (light-producing) half reaction in the beetle^23^ is replaced by a methionine in *Arachnocampa*. We are now working on producing the *A. luminosa* luciferase in a recombinant form, to be assayed for bioluminescent activity using the native glowworm luciferin substrate as described in this report. Initial attempts at expression in multiple standard prokaryotic strains, as well as in insect and human cells, have so far not yielded soluble protein, but continued efforts in this regard are ongoing.

### Xanthurenic acid (XA) and tyrosine (Tyr) contribute to glowworm bioluminescence

**Figure 4:**
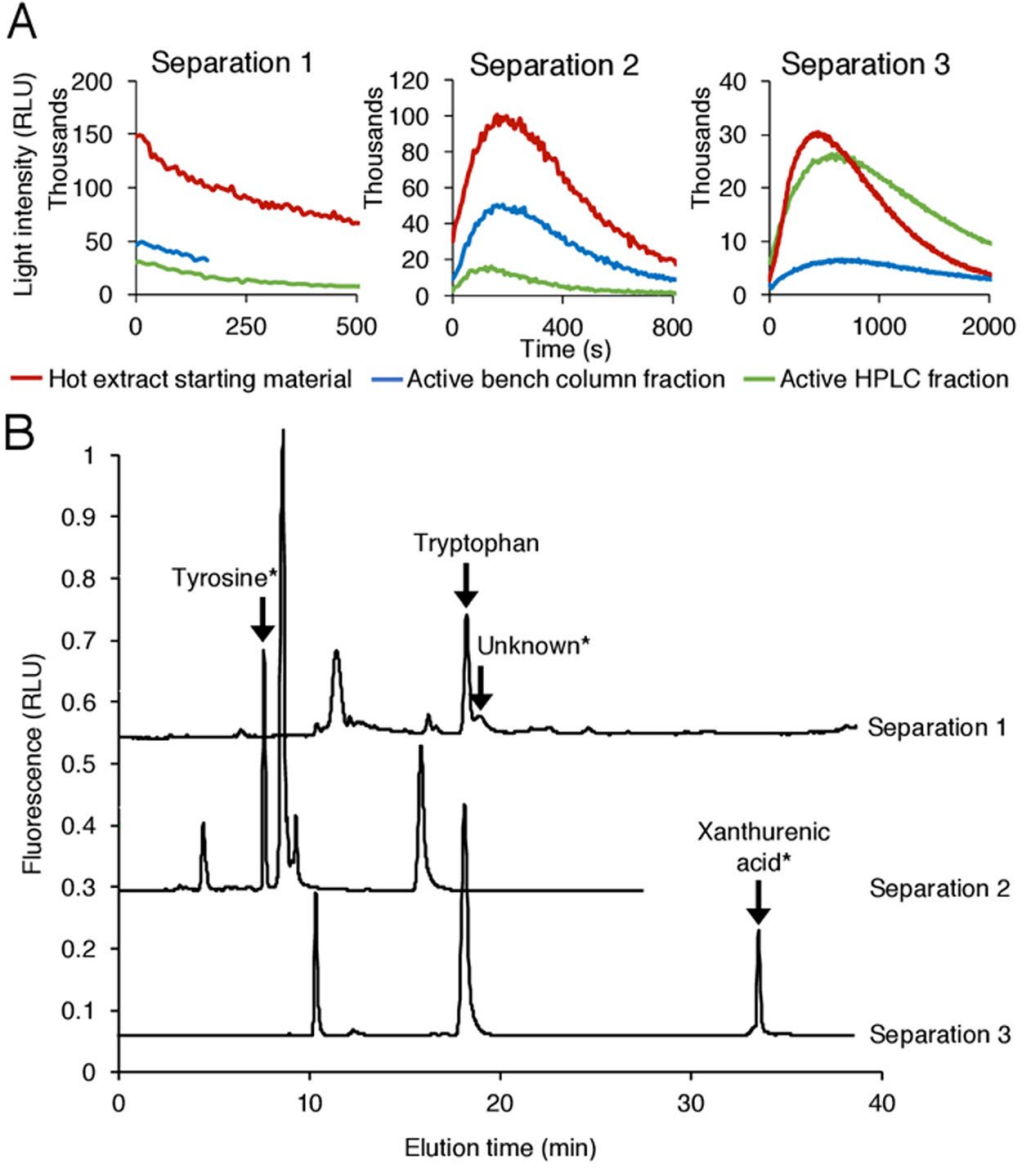
Isolation of Tyr and XA from *A. luminosa* hot extract. (**A**) Time courses of luminescence reactions with luciferin-containing fractions from chromatographic separations of *A. luminosa* hot extracts. In each assay, material to be assayed was added to crude luciferase, and ATP-Mg^2+^ was injected 5 s before start of measurement. (**B**) Chromatogram showing the fractionation of three preparations from *A. luminosa* hot extract by semi-preparative HPLC, monitored by fluorescence (excitation 280 nm, emission 415 nm). Peaks correlating with fractions that luminesced when added to crude luciferase-ATP-Mg^2+^ are indicated by *. Back injection of these fractions produced the same peak. Separation 1 produced light with FP profile; separations 2 and 3 produced light with SP profile. RLU: relative luminescence units.

Initial attempts at isolating the glowworm luciferin were carried out on *A. luminosa* hot extract preparations using reverse phase (C18) bench column chromatography followed by reverse phase HPLC (high pressure liquid chromatography). Separations from three different hot extract preparations are reported here, carried out using slightly different elution strategies (Supplementary Table S4) and with hot extracts that exhibited different luminescent time course profiles (Fig. 4A). Fractions eluted from the bench column were concentrated and then tested for the presence of luciferin. Fractions that produced luminescence were further separated by HPLC. Eluted HPLC fractions were also concentrated and tested for bioluminescent activity; any of these fractions demonstrating luminescence were then characterised by LC-MS (liquid chromatography-mass spectrometry). Separation, concentration and handling steps were carried out under N_2_ to minimise oxidation. Time course profiles for the starting material and luminescent fractions isolated for each separation are provided in Fig. 4A, and chromatograms for each semi-preparative HPLC fractionation are provided in Fig. 4B. LC-MS analysis details can be seen in Supplementary Fig. S8.

These three separations each led to the isolation of different components that produced light when assayed. Separation 1 yielded a fraction that eluted from the C18 bench column with 20% acetonitrile that exhibited an FP type luminescence reaction time course, and was selected for further fractionation by HPLC. The active fraction eluting during HPLC also produced FP luminescence, and corresponded to a peak that eluted immediately after tryptophan. The compound in the active peak could not be identified due to a lack of material. Further attempts to purify this compound by reverse phase HPLC produced no bioluminescent fractions. From separation 2, a luminescent fraction (SP-type) from the bench column was obtained following elution with water. This fraction was injected onto the HPLC column to give an early-eluting fraction that also produced SP luminescence. LC-MS analysis of this fraction showed that it mainly contained Tyr, with small amounts of 3-OH kynurenine and leucine. During separation 3, a fraction was eluted from the bench column with 20% acetonitrile that produced SP type luminescence. Following HPLC purification, a late-eluting SP luminescent fraction was obtained. LC-MS analysis showed that this fraction mainly contained XA, with trace amounts of riboflavin.

**Figure 5:**
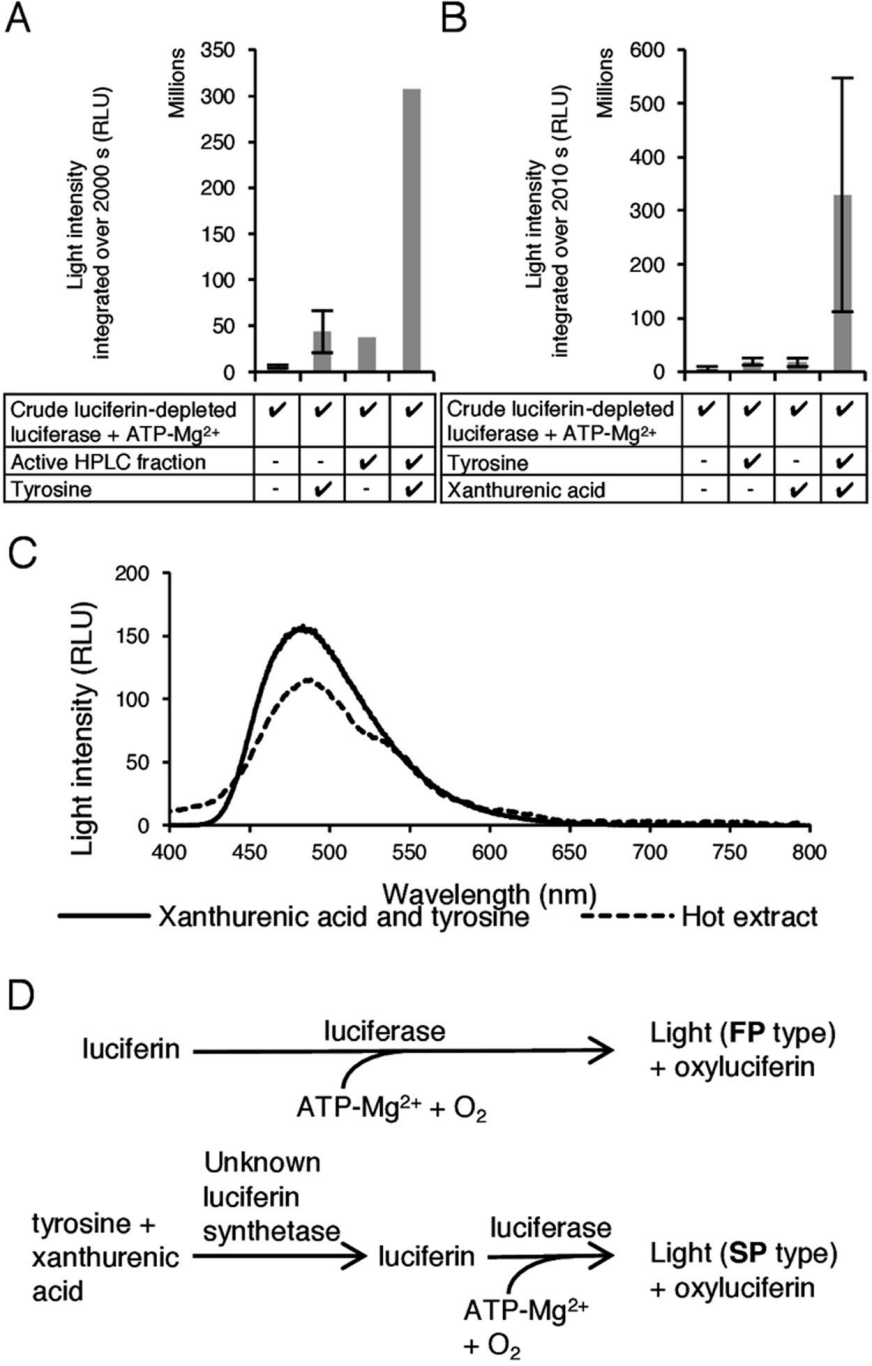
XA and Tyr produce light when added to crude luciferin-depleted *A. luminosa* luciferase. ATP-Mg^2+^ was injected 5 s prior to measurement beginning for all assays. RLU: relative luminescence units. (**A**) Active HPLC fraction from separation 3 mixed with Tyr produces more light than either alone when added to crude luciferin-depleted luciferase. (**B**) XA mixed with Tyr produce more light than either alone when added to crude luciferin-depleted luciferase. Where error bars are present, data points represent the mean of two **(A)** or three **(B)** experiments; error bars indicate standard deviation. No error bars indicate that replicate assays were not carried out due to lack of material. (**C**) Luminescence emission spectra of either hot extract or XA and Tyr added to crude luciferin-depleted luciferase and ATP-Mg^2+^. (**D**) A proposed model of glowworm luciferin metabolism. The luminescence time course profile produced (FP type or SP type) is dependent on the starting material provided.

Since Tyr and XA were isolated in these separations, and appeared to be associated with luminescence activity, we decided to assay the combination of pure (commercial) L-Tyr and the active HPLC fraction containing XA from separation 3 with the crude luciferase. This combination produced 8-fold greater luminescence than XA-containing fractions tested without additional Tyr (Fig. 5A). Next, we assayed pure L-Tyr plus pure (commercial) XA for luciferin activity using our assay (Fig. 5B). Both L-Tyr and XA separately produced luminescence above that of the crude luciferase alone, but when they were assayed together, the light produced was about eighteen-fold more intense. These results suggest that Tyr and XA are both required for glowworm bioluminescence. Furthermore, LC-MS analysis of crude luciferase preparations indicated that both compounds are present (Supplementary Fig. S4 and Table S1), which could explain why luminescence is also observed when Tyr or XA were tested alone.

The bioluminescence emission spectrum produced by the reaction of XA and Tyr with luciferin-depleted luciferase and ATP-Mg^2+^ has the same peak wavelength as that produced by the reaction of hot extract with luciferin-depleted luciferase and ATP-Mg^2+^ (Fig. 5C), consistent with this being the same bioluminescent reaction.

Intriguingly, all of these assays produced light which took many minutes to reach peak intensity (SP type time course). One explanation for this could be that Tyr and XA are precursors of the actual glowworm luciferin, and the delay in peak light intensity is due to the extra time required to conjugate the compounds together into a luciferin by an unknown luciferin synthetase enzyme present in the crude luciferase preparation, before the bioluminescence reaction can occur. If this theory is correct, the addition of a ‘fully formed’ *Arachnocampa* luciferin to the crude luciferase would produce rapidly peaking light intensity (FP type). Therefore, the compound isolated during separation 1 that produced a FP type luminescent time profile (but was unable to be identified by LC-MS) may be the glowworm luciferin. A schematic of the putative two-step process that Tyr and XA require to produce SP type bioluminescence is provided in Fig. 5D. A similar two-stage bioluminescence scheme was proposed by Airth and Foerster for bioluminescent fungi^26^, and recently confirmed by Purtov *et al*., who were also able to identify the luciferin precursor as hispidin and the luciferin as 3-hydroxy hispidin^27^.

### Isotope labeling identifies a glowworm luciferin-related compound (LRC) comprising tyrosine and xanthurenic acid

Since the actual *A. luminosa* luciferin proved difficult to isolate and identify from hot extract, and we now suspected that Tyr and XA were precursors to the *A. luminosa* luciferin, we decided to try extracting the luciferin or luciferin-related compounds (such as a luciferin precursor or the end product of the bioluminescence reaction) from crude luciferase preparation supplemented with Tyr and XA. We have shown above that such preparations appear to contain the necessary constituents, likely to be enzyme(s), required for luciferin synthesis.

To help identify such compounds, the Tyr used consisted of a mixture of isotope-labeled Tyr (^13^C_9_H_11_^15^NO_3_) and unlabeled Tyr (^12^C_9_H_11_^14^NO_3_) in a 1:1 ratio. Any Tyr metabolites biosynthesised from this mixture would then exist as a pair of compounds, with identical LC-MS elution times but different masses, one made from labeled Tyr and one made from unlabeled Tyr. Such pairs can be searched out automatically in LC-MS data (SIEVE ‘perfect pair’ program, Thermo Fisher Scientific). Any such Tyr metabolite found only in reactions which luminesce is likely to be the luciferin or a luciferin-related compound.

**Figure 6:**
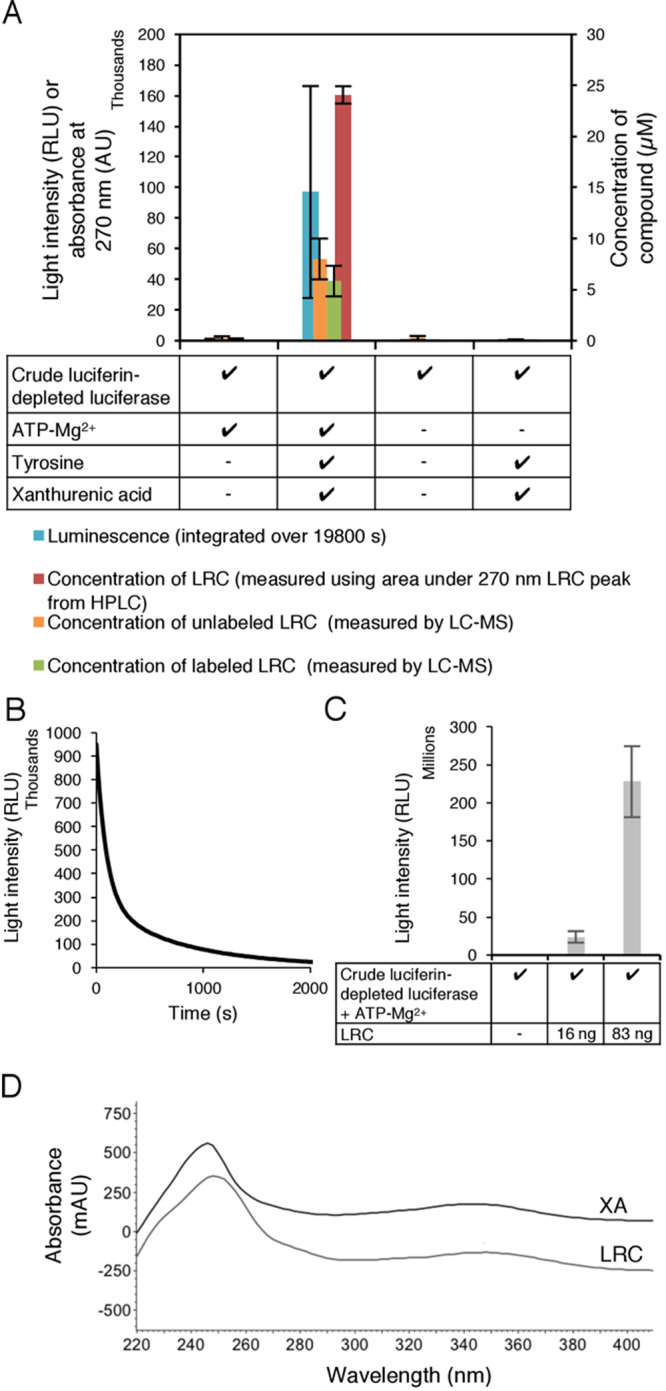
Isolation and identification of LRC. (**A**) Bioluminescence and the compound LRC are only produced when Tyr and XA are added to a reaction mix of luciferin-depleted crude luciferase and ATP-Mg^2+^. LRC concentration was measured using LC-MS and HPLC-UV. Data points represent the mean of three experiments; error bars indicate standard deviation. (**B**) Isolated LRC produces light with an FP time course when added to luciferin-depleted crude luciferase. ATP-Mg^2+^ was injected 5 s prior to measurement. (**C**) Isolated LRC produces light in a concentration-dependent manner. An increase in LRC added to the assay of about 5-fold resulted in an increase of light of nearly 10-fold. Data points represent the mean of three experiments; error bars indicate standard deviation. (**D**) Comparison of UV-Vis spectra of XA and LRC. Absorbance maxima: 248 and 348 nm for LRC, 244 and 348 nm for XA. RLU: relative luminescence units.

We therefore prepared four reaction mixtures: (1) crude luciferase, labeled and unlabeled Tyr, XA and ATP-Mg^2+^; (2) crude luciferase, labeled and unlabeled Tyr, and XA but no ATP-Mg^2+^; (3) crude luciferase and ATP-Mg^2+^; and (4) crude luciferase alone. The reactions were incubated for 5.5 hours and light production recorded before being prepared for LC-MS analysis using HPLC. Reaction mixture 1 contained one main ‘perfect pair’: co-eluting compounds with MH^+^ 369.1080 Da and 379.1350 Da, a mass difference indicating incorporation of C_9_N from Tyr (Supplementary Table S5). This pair of compounds was only produced in the reaction mixture that produced luminescence, reaction mixture 1 (Fig. 6A).

In other experiments, either only labeled or only unlabeled Tyr was mixed with crude luciferase, XA and ATP-Mg^2+^ (Supplementary Fig S9). Both mixtures produced luminescence, but the unlabeled Tyr gave only the compound with MH^+^ 369.1080. Labeled Tyr gave the compound with MH^+^ 379.1350 Da, plus a small amount of MH^+^ 369.1080 Da compound, probably from the unlabeled Tyr in the crude luciferase. This is referred to as glowworm luciferin-related compound (LRC).

In order to produce enough LRC for further characterisation, we scaled up the preparation with unlabeled Tyr, and isolated about 10 µg of material. Initially this batch of LRC was 99% pure (by HPLC-UV and LC-MS), but it was unstable, giving oxidised products on storage. Portions of purified LRC were added to luciferin-depleted crude luciferase and ATP-Mg^2+^ to assay for bioluminescent activity. LRC produced intense FP type luminescence (Fig. 6B), which appears to be concentration-dependent (Fig. 6C). This evidence suggests that LRC is either the *A. luminosa* luciferin or a luciferin precursor.

### Characterisation of luciferin-related compound (LRC)

MS and MS fragmentation (MS^2^) analyses were carried out with unlabeled and stable isotope labeled LRC using LC-MS (Supplementary Table S5). High-resolution MS revealed that unlabeled and labeled LRC had molecular formulae of C_19_H_16_N_2_O_6_ and C_10_^13^C_9_H_16_N^15^NO_6_, respectively, consistent with compounds containing either unlabeled or labeled Tyr, plus XA, minus H_2_O. This is most readily explained by LRC having an amide or an ester linkage between Tyr and XA moieties. The similarity of the UV-Vis spectra of LRC and XA (Fig. 6D) show that the XA chromophore was retained in LRC and that the ring was not further oxidised or reduced.

MS^2^ of LRC gave fragments also observed in the MS^2^ of XA and Tyr, as well as four further fragments that incorporated both XA and tyrosine moieties (Supplementary Table S5). The lack of an intact [Tyr + H]^+^ ion indicates that these two moieties are not linked by a weak phenol ester (depside bond)^28^. Unlabeled LRC showed an initial loss of CO_2_H_2_, while labeled LRC [M + H]^+^ showed a corresponding initial loss of ^13^CO_2_H_2_ (Supplementary Table S5). This indicated that ^Tyr^carboxylic acid was lost from LRC as formic acid without the cleavage of the XA residue from the Tyr residue, thus showing that Tyr was not attached to XA via the ^Tyr^carboxylic acid. The presence of the [M-CO_2_H_2_-H_2_O]^+^ ion is best explained by loss of water from the [M-CO_2_H_2_]^+^ ion; [XA + H]^+^ also fragments via the loss of water (m/z 206–188, Supplementary Table S5). The C_10_^Tyr^CH_9_N^Tyr^NO_3_ ion meanwhile is likely formed via the cleavage of most of the tyrosyl residue from the XA leaving only a ^Tyr^C and ^Tyr^N attached, while the C_10_H_8_NO_4_^+^ ion likely represents the [XA + H]^+^ ion or similar.

The LRC NMR (Nuclear magnetic resonance) spectrum showed signals similar to those of the 5, 6 and 7 protons of XA and the 2 + 6, 3 + 5 and *β* protons of Tyr, suggesting that these regions are similar in LRC. The XA H3 signal (singlet at 7.8 ppm) had no near equivalent in the spectrum of LRC, but there was a one-proton singlet at 9.1 ppm in the LRC NMR spectrum, absent from Tyr and XA spectra. ^13^C and 2D NMR spectra could not be collected on this sample due to the small amount (10 µg) available; the proton NMR took over a day to collect.

**Figure 7:**
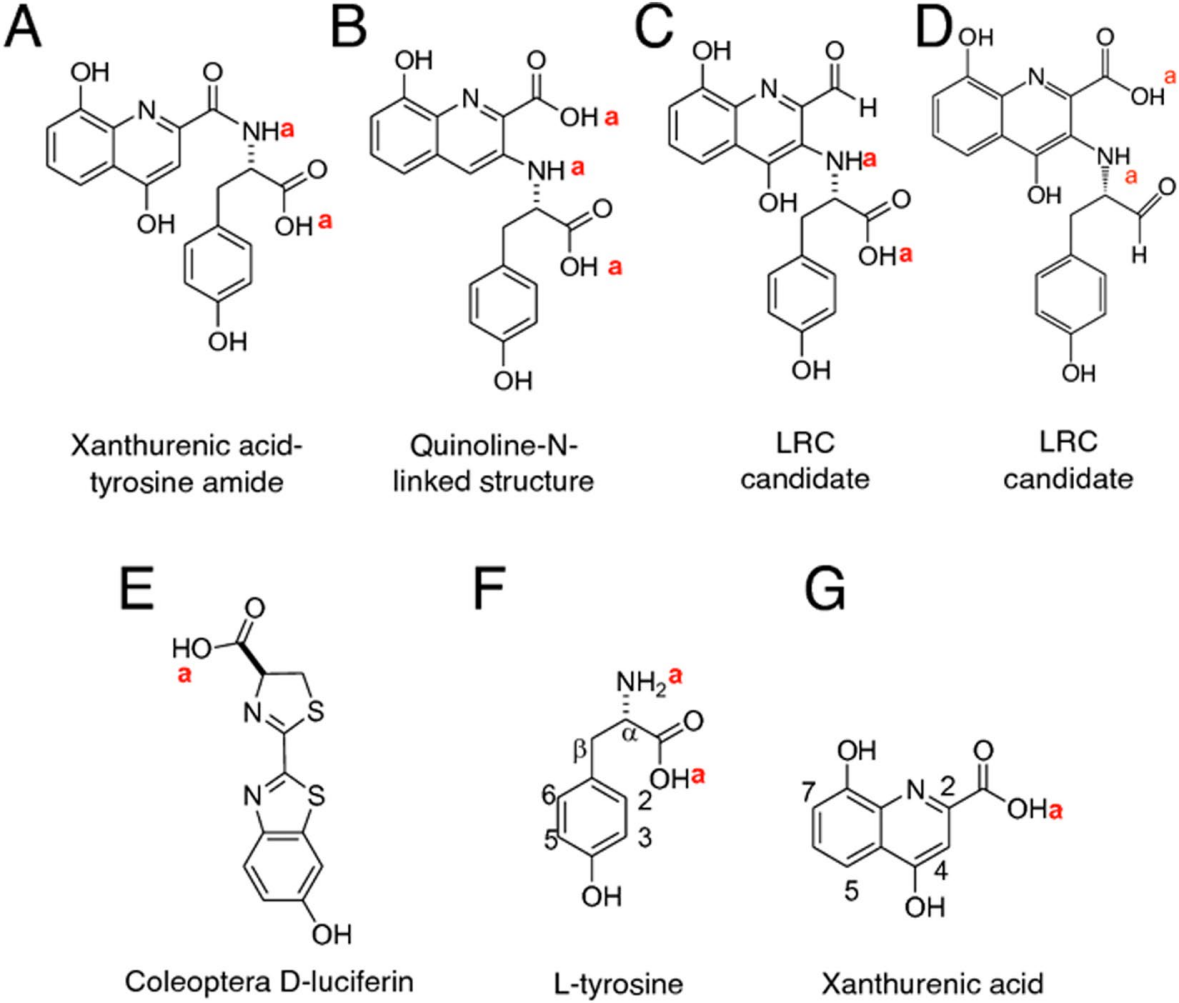
Small molecules involved in the bioluminescence of the glowworm and the firefly. (**A**) Possible XA-Tyr amide. (**B**) Possible quinoline-N-linked structure. (**C**), (**D**) Candidate structures of *A. luminosa* LRC. (**E**) Coleoptera D-luciferin, found in fireflies, click beetles and railroad worms. (**F**) L-Tyr. (**G**) XA. “a” indicates protons exchangeable with deuterium under acid conditions.

The only possible amide link, not involving a ^Tyr^carboxylic acid linkage, is that of ^XA^carboxylic acid to ^Tyr^N amide (Fig. 7A). However, such a structure does not explain the high chemical shift singlet at 9.1 ppm (Supplementary Table S6 and Fig. S10). The few known amides involving a ^XA^carboxylic acid show ^XA^H3 around 7.3 ppm^29^, effectively ruling out the structure Fig. 7A. The α proton in LRC is at 5.4 ppm, compared to 4.5 ppm in Tyr (Supplementary Table S6), suggesting linkage of XA to Tyr via the ^Tyr^N group (given that the ^Tyr^carboxylic acid is intact, see above, and N-phenyl Tyr has its *α* proton deshielded compared to Tyr)^30^. Since we already dismissed the XA -Tyr amide, Fig. 7A (see above), we considered compounds with ^Tyr^N attached directly to the XA quinoline ring at C3 or C4, with loss of the 4OH, i.e. the structure in Fig. 7B, or the C4-N isomer. However, direct infusion MS analysis of LRC dissolved in 1:1 D_2_O:CD_3_CN with 0.1% formic acid showed [MD]^+^ ions up to 372 Da, compared to [MH]^+^ of 369 Da for unlabelled LRC (Supplementary Table S7). LRC therefore contains two protons that can exchange with deuterium under acidic conditions (excluding the ionising proton required for [M + H]^+^). In contrast (excluding ionising protons), XA contains one acid exchangeable proton (COOH) while Tyr has three (NH_2_ and COOH). This shows that the XA and Tyr phenols are not exchangeable under the conditions, and that structure Fig. 7B should contain three acid-exchangeable protons (excluding the ionising proton). Since LRC contains two acid exchangeable protons (excluding the ionising proton), structure Fig. 7B was dismissed.

In light of this evidence, we propose that the 9.1 ppm singlet in the ^1^H NMR spectrum of LRC (Supplementary Table S6) is caused by an aldehyde proton, suggesting that the candidate luciferin has the structure in Fig. 7C or in 7D. Model compounds show singlet aldehyde proton signals at about 10 ppm^31^ or 9.6 ppm^32,33^. Both these values are a little higher but they do indicate that an aldehydic proton could be responsible for the 9.1 ppm peak. The Fig. 7D structure may appear prohibited due to the lack of coupling between the aldehyde proton and the vicinal Tyr α proton. However, some similar aldehyde protons exhibit singlet ^1^H NMR peaks at 9.6 ppm^32,33^.

The proposed structure in Fig. 7C is preferred because it contains a free ^Tyr^carboxylic acid, which makes the ^Tyr^CO_2_H_2_ MS loss easy to rationalise. This structure in Fig. 7C is still problematic, because it is difficult to determine the origin of the C_10_H_8_NO_4_^+^ ion [XA + H]^+^. However, some aldehydes with a vicinal group that can hydrogen bridge to the aldehyde (e.g. formylchromones and 3-formylcoumarins) do form fragment ions during electrospray ionisation MS^2^ in which the aldehyde is converted into a carboxylic acid^34^. The carboxylic acid group is thought to be produced from the aldehyde via loss of H_2_ followed by water adduction within the collision cell. Such a process occurring to a suitable LRC fragment ion could possibly result in the [XA + H]^+^-like ion.

We therefore propose the LRC structure shown in Fig. 7C. To confirm this hypothesis, synthesis of the proposed LRC structure is being pursued, to be followed by spectral comparisons and bioluminescence assays.

## Discussion

The purification and characterisation work reported above has shown directly that the *A. luminosa* glowworm luciferase is the 59 kDa protein encoded by transcript 62762, which we had identified previously in differential expression analyses^17^. Since the glowworm bioluminescence reaction requires ATP, it makes sense for the glowworm luciferase to be encoded by a member of the ANL superfamily of adenylating enzymes, which all require ATP for catalysis. The larval serum proteins and the aldehyde dehydrogenases found in the active fraction are unlikely to catalyse the glowworm reaction because such proteins are not known to require ATP. Because of the difficulty of collecting *A. luminosa* larvae, we are working on producing this protein recombinantly. It will then be assayed for bioluminescent activity using the native glowworm luciferin substrate described here.

The combination of XA and Tyr proposed here for glowworm luciferin formation is unprecedented in the luciferins of other bioluminescent systems^10,11^. The other known insect luciferin, from the bioluminescent Coleoptera, i.e. fireflies, click beetles and railroad worms, Fig. 7E, is biosynthesised from 1,4-hydroquinone and two L-cysteines^35^. XA and Tyr are reasonable precursors for the glowworm luciferin for several reasons. Tyr (Fig. 7F) is a component of both coelenterazine and of *Fridericia heliota* luciferin^36,37^, and is also an important insect metabolite^38–43^ that can undergo a number of radical, redox and charge transfer processes^44–47^. XA (Fig. 7G) is a well-studied insect metabolite^48–50^ and metabolic precursor^51–53^. It is an antioxidant free radical scavenger, used by insects to prevent damage by reactive oxygen species^54,55^. Furthermore, like other quinolines, XA is chemiluminescent when oxidised by free radicals^54,56^. There is evidence of XA synthesis in *A. luminosa* light organs: in our study 3-OH kynurenine (converted into XA in insects by kynurenine transaminase)^54^ was found in the *A. luminosa* light organ (Supplementary Figs S4, S8, Table S1); and we previously observed that kynurenine transaminase is more highly expressed in the *A. luminosa* light organ compared with the rest of the larval body^17^ (although not to a statistically significant extent).

To confirm the proposed LRC structure shown in Fig. 7C, synthesis is being pursued, to be followed by spectral comparisons and bioluminescence assays.

The LRC molecule isolated in these experiments is likely to be the *A. luminosa* luciferin, or rapidly converted to it, because LRC produces high intensity, fast peaking blue-green luminescence when mixed with crude luciferin-depleted luciferase and ATP-Mg^2+^. However it is not the same luminescent compound that we isolated from the glowworm (see Fig. 4B) as these molecules showed different retention times. We cannot rule out the possibility that LRC is a precursor to the *A. luminosa* luciferin, converted by an unknown enzyme from the light organ lysate into the active substrate.

Our findings contrast with those of Trowell *et al*.^18^ who recently reported that recombinant *A. richardsae* luciferase uses firefly D-luciferin to produce blue light. In *A. luminosa*, we could not detect a cross reaction between firefly and *A. luminosa* molecules (Fig. 2). Further, we did not detect any firefly D-luciferin in *A. luminosa* lysate (Supplementary Fig. S4 and Table S1). Our results are in agreement with Lee^13^, who reported that firefly luciferin did not stimulate light production when added to reconstituted lysate made from freeze-dried *A. richardsae* light organs. We note that the bioluminescent emission spectrum reported by Trowell *et al*. from recombinant luciferase and firefly D-luciferin^18^ is very much broader than that reported by Lee when adding hot extract made from *A. richardsae* light organ lysate to crude luciferase made from *A. richardsae* light organ lysate^13^. Therefore, either *A. luminosa* and *A. richardsae* use different luciferins, or the luminescence reported by Trowell *et al*. stems from another source. *A. richardsae* luciferase may be able to produce light with firefly luciferin, since even acyl-CoA enzymes from non-bioluminescent insects have been found to produce some light with firefly luciferin or an analog^57,58^. It would be useful to compare the bioluminescence Trowell *et al*. reported with that produced when adding hot extract made from *A. richardsae* light organ lysate to firefly luciferase and to crude *A. richardsae* luciferase (as in Fig. 1A).

It was previously assumed that evolutionarily unrelated bioluminescent systems use luciferase enzymes with very different structures and mechanisms, evolved from different enzyme families^1,7,59^. There are also a variety of luciferin substrates^1,11^, although species with evolutionarily unrelated bioluminescent systems sometimes share a luciferin, especially when a luciferin is readily available from the food chain. For example, the Japanese firefly squid, some fish, Hydrozoa, and Anthozoa all use coelenterazine or coelenterazine derivatives as a luciferin substrate^60,61^, which they do not synthesise, instead feeding upon coelenterazine-producing marine crustaceans such as the shrimp *Systellaspis debilis*^62^ and the copepod *Metridia pacifica*^37^. In the *A. luminosa* bioluminescent system, we see the trend reversed: it has a novel luciferin not used by any other characterised system, and a luciferase belonging to an enzyme family seen already in the bioluminescent beetles (including fireflies). In fact, there is a growing group of bioluminescent systems that are evolutionarily distinct and are known to require ATP to produce light, but use entirely different luciferins. Three of these systems use enzymes related to acyl-CoA synthetases to carry out bioluminescence: the bioluminescent beetles/firefly^63^, the Japanese firefly squid (*Watasenia scintillans*)^64–66^, and now the *Arachnocampa* glowworm. The Siberian earthworm (*Fridericia heliota*) also uses ATP and an entirely different luciferin to produce light^67,68^; we await further research to see if the *F. heliota* system also uses an enzyme related to acyl-CoA synthetases. Recently, another possible example of convergent evolution of similar enzymes into luciferases has been identified. A luminous echinoderm, the ophiuroid *Amphiura filiformis*, appears to have a luciferase homologous to that of the sea pansy *Renilla* (Cnidaria)^69^. It is therefore evident that these bioluminescent systems are an example of evolved functional convergence of enzymes at the molecular level. It appears that the ability of the acyl-CoA synthetase enzymes to adenylate their substrate molecules provides them with the capacity to evolve bioluminescent activity using a range of substrates.

In conclusion, this investigation has revealed key components of the bioluminescent system of the New Zealand glowworm. The purified luciferase enzyme is from the same adenylating enzyme superfamily as firefly luciferase. Two *A. luminosa* light organ metabolites, Tyr and XA, form a luciferin candidate compound which produces light when added to a crude *Arachnocampa* luciferase. Neither the proposed luciferin candidate structure nor similar structures have been reported from any other bioluminescent organism. This novel glowworm bioluminescent system could be suitable for the development of new research tools and analytical methods.

## Methods

### Sample collection

*A. luminosa* is an endemic species of Aotearoa/New Zealand, therefore Māori were consulted about this research through Te Komiti Rakahau ki Kāi Tahu (the Ngāi Tahu Research Consultation Committee). Glowworms were collected at night from locations including Dunedin, Te Anau, Palmerston North and Auckland (voucher specimens of each collection have been stored in 75% ethanol, details are available from the authors). Live glowworms were snap frozen on foil placed on dry ice, and stored at −80 °C until required. Using a razor, light organs were removed from the glowworm bodies while still frozen, taking care to excise only white light organ tissue, and not the darker non-light organ tissue.

### Chemicals

Stable isotope-labeled L-Tyr (^13^C_9_H_11_^15^NO_3_; 99% pure) was purchased from Cambridge Isotope Laboratories. Unlabeled L-Tyr, XA and other amino acids were purchased from Sigma Aldrich and used without further purification. Labeled and unlabeled Tyr and XA were dissolved in NaHCO_3_ (1.2 mg/ml) before use. Water (Type 1) was produced using a Milli-Q ultrapure system. LiChrosolv LC grade solvents were purchased from Merck and were used for all chromatography steps.

### Production of lysate, hot extract and crude luciferin-depleted luciferase

*Lysate:* frozen light organs (usually 20–40 or 0.023–0.107 g) were homogenised in a B type glass douncer (ThermoFisher Scientific) with 1–4 ml of 100 mM MOPS pH 7.4, 2 mM DTT (unless mentioned otherwise) on ice. The lysed solution was centrifuged at 15800 rcf for 5 min at 4 °C to remove cell debris and the supernatant retained. *Hot extract:* lysate was heated at 95 °C for 5 min, then centrifuged at 15800 rcf for 5 min at 4 °C to remove denatured protein and the supernatant retained. *Crude luciferin-depleted luciferase:* a Zeba^TM^ spin 2 ml desalting column (ThermoFisher Scientific) was equilibrated by centrifugation using 100 mM MOPS pH 7.4, 2 mM DTT. Lysate as prepared above was loaded onto the spin column and the column centrifuged for 1 min, eluting crude luciferin-depleted luciferase. Lysate, hot extract and crude luciferin-depleted luciferase preparations were either used immediately or stored at –80 °C and thawed on ice before use.

### Bioluminescence assays

Assays were carried out in white, flat bottomed 96 well plates (Nunc©, ThermoFisher Scientific). Typically, assays included 50–60 µl of assay buffer (100 mM MOPS pH 7.4, 2 mM DTT, unless mentioned otherwise), 5 µl of hot extract and 5 µl of crude luciferin-depleted luciferase preparation. ATP-Mg^2+^ solution (40 mM ATP, 80 mM MgSO4, 0.1 M MOPS pH 7.4, 2 mM DTT) was then added simultaneously to all wells being assayed (30 µl per well) using a multi-channel pipette, assay reactions mixed, and monitoring of light intensity of each well started immediately afterward. Light intensity was measured using a microplate reader in luminescence mode; either the POLARstar (BMG Labtech) (gain 4095, measurement time for each well within a cycle was 0.58 s with an overall cycle time of 7 s for the measurement of 12 wells) or the CLARIOstar (BMG Labtech) (gain 3500, focal height 11 mm, rapid speed function, measurement time for each well within a cycle was 0.42 s with an overall cycle time of 5 s for the measurement of 12 wells). Luminescence of samples tested non-concurrently or with different crude luciferin-depleted luciferase preparations could not be compared directly, because crude luciferase activity was highly variable between different preparations, and because the activity of any preparation reduced over time. Assay mixtures used to assess the effect of bioluminescence included either 50 µl L-Tyr solution (0.94 µg/ml) and/or 50 µl XA solution (58 µg/ml).

Assay results were analysed using Microsoft Excel. Light intensity in relative luminescence units (RLU) were integrated between each time point using the trapezoid method [(time A − time B) * (height A + height B)/2] and the results summed over the time of interest. A sample was considered to contain luciferin substrate (or luciferase enzyme) if the integrated light intensity over a set time for the sample mixed with crude luciferin-depleted luciferase (or hot extract) and ATP-Mg^2+^ was at least three times the integrated light intensity for a crude luciferin-depleted luciferase and ATP-Mg^2+^ only control, tested at the same time under the same conditions. Statistical analyses: error bars show one standard deviation above and below the mean (average of three replicate reaction mixtures unless stated otherwise). Statistical significance was tested using a student T test (two tailed, two sample unequal variance).

Bioluminescence emission spectra were measured using a Cary Eclipse fluorescence spectrophotometer (Varian) in chemiluminescence mode from a reaction mixture in a 100 µl or 1 ml cuvette (settings: gate time 200 ms, scan mode, emission 400–600 nm, slit 20 nm, data interval 0.1500 nm, emission filter open, PMT voltage high, filter size 101, temperature 10 °C).

### Fast protein liquid chromatography (FPLC)

A Superose 6 10/300 GL column (approximately 24 ml; from Pharmacia, now GE Healthcare) was used for gel filtration, and a 1 ml Mono Q 5/50 GL column (GE Healthcare) was used for ion exchange on an Äkta Purifier FPLC system (GE Healthcare). Prior to purification, columns were equilibrated with 4–6 column volumes of buffer A (30 mM MOPS pH 7.5, 10% glycerol, 1 mM EDTA, 2 mM DTT). Flow rates were 0.4 mL/min for gel filtration and 0.5 ml/min for ion exchange. 1 mL fractions were collected and 5 µl of each fraction assayed for luciferase activity using 5 µl of a hot extract preparation as substrate. Gel filtration was carried out on 2 ml of glowworm light organ lysate, part of a 4 ml batch previously prepared using 0.753 g of glowworm light organ tissue from 186 glowworms, and stored at −80 °C. Before chromatography, the 2 ml lysate aliquot was thawed on ice, filtered through a 0.2 µm filter and assayed to check for bioluminescent activity before being loaded onto the gel-filtration column. Fractions with bioluminescent activity eluting from the gel filtration column were pooled and injected onto the ion-exchange column, which was then washed with 5 column volumes of buffer A, and programmed to carry out elution gradient steps of 0–20% buffer B (30 mM MOPS pH 7.5, 10% glycerol, 1 mM EDTA, 2 mM DTT, 1 M NaCl) over 20 ml, and 30–100% buffer B over 0 ml. Calibration standards (Sigma) were loaded onto the gel filtration column under the same conditions as for the glowworm lysate.

### Protein identification and functional annotation

#### SDS-PAGE analysis

200 µl of each fraction eluted from FPLC was precipitated by adding 800 µl of acetone chilled to −20 °C, then centrifuged at 16,100 rcf for 10 min, the supernatant discarded and pellet left at 37 °C for 20 min to dry. The pellet was resuspended in 10 µl of loading buffer containing β-mercaptoethanol before being loaded onto the gel. Samples were run on Mini-PROTEAN TGXTM 4–20% polyacrylamide precast SDS-PAGE gels (BioRad) and stained using Coomassie blue. Gels were scanned using a Gel DocTM XR + system (BioRad), and molecular weight estimates for gel bands were calculated using Image Lab software version 5.0 (BioRad).

#### MS analysis

Each band or segment of interest was excised from the SDS-PAGE gel, subjected to tryptic digestion, and peptides analysed on a 4800 MALDI tandem Time-of-Flight Analyzer (MALDI TOF/TOF, Applied Biosystems, MA) at the Centre for Protein Research (University of Otago, Dunedin, New Zealand). The resulting peptide mass data were searched against the combined glowworm transcriptome assembly translated into all possible reading frames by Proteome Discoverer 1.4, using the Mascot search engine (http://www.matrixscience.com). Identified proteins were annotated by searching the GenBank non-redundant database at the NCBI (http://www.ncbi.nlm. nih.gov) using the BLASTX software^22^ with default parameters. An E-value cut-off of 1E-6 was used to identify similar annotated proteins from which function could be inferred.

### Bench column and high pressure liquid chromatography (HPLC)

#### Instrumentation

Agilent 1100 with photodiode array and Agilent 1260 Infinity with fluorescence detector, UV-Vis detector and automatic fraction collection unit. Columns: Phenomenex Synergi 4 µM Hydro RP 80 Å: analytical (150 × 4.6 mm) and semi-preparative (150 × 10 mm). *Mobile phase:* A, H_2_O; B, acetonitrile. *HPLC method 1:* 0% B–6% B over 5 min, 6% B for 23 min, 6–25% B over 14 min, 25–100% B over 10 min, 100% B for 4 min, 100–0% B over 1 min, 0% B for 5 min. *HPLC method 2:* 0% B–6% B over 5 min, 6–100% B for 10 min, 100% B for 7 min, 100–0% B over 1 min, 0% B for 5 min with A and B both containing 0.1% formic acid. *Flow rate:* 0.5 ml/min for hydro C18 analytical column, 2.3 ml/min for hydro C18 semi-prep column. *Fluorescence detection:* excitation 280 nm, emission 415 nm. *UV Detection:* 210, 250, 270, 280, 300, 320, 350, 380 and 400 nm. The identities of compounds eluted using HPLC were confirmed by comparison to purchased standards analysed using LC retention time, MS, MS2 and UV-Vis spectra.

#### Anaerobic technique

Most handling of *A. luminosa* extracts during bench chromatography and HPLC was done under N_2_ in a glove bag. When this was not possible, samples were kept in vials under N_2_. Liquids were transferred between vials using a gas tight Hamilton syringe. All solvents were degassed by bubbling N_2_ through the sample. Samples were concentrated by introducing frozen samples (−80 °C) to a desiccator inside a N_2_ filled glove bag. The desiccator was then sealed, removed from the glove bag and attached to high vacuum until the samples were dried. Samples were kept under vacuum until the desiccator was opened inside a N_2_-filled gas bag. They were then dissolved in degassed solvent and refrozen using dry ice.

#### Chromatography

Each lysate, crude luciferase and hot extract preparation used was first assayed for luminescent activity. Hot extract was then loaded onto a pre-equilibrated 0.5 g (unless stated otherwise) Isolute C18 column under N_2_, eluted with a gradient of water to acetonitrile. Fractions were eluted using positive N_2_ pressure. Eluted fractions were frozen on dry ice, concentrated using the method described above, then dissolved in water or buffer (0.1–1 ml), and samples assayed for the presence of luminescent compounds. Fractions with luminescent activity were combined, concentrated, and then further separated using HPLC method 1 under a N_2_ atmosphere. HPLC fractions were also concentrated and assayed for luminescence activity.

#### Luminescence yield

The light intensity integrated over a selected time was compared between eluted fractions and the original column input material and hot extract and these values, along with concentration and subsampling factors, were used to determine the luminescence yield compared with the initial hot extract. Fractions were also analysed using HPLC method 1.

### Isolation of LRC for stable isotope labeling analysis

Four reaction mixtures were prepared in triplicate as described in Results. The reactions were incubated inside the Clariostar well plate luminometer at room temperature for 5.5 h and light production measured. In preparation for LC-MS analysis, each reaction mixture was added to a pre-equilibrated Sorenson Bioscience Multiguard II Filter tip containing 30 mg of C18 resin. The filter tips were positioned in holes drilled through the lids of 1.5 ml plastic tubes. Centrifugation was then used to elute reaction mixtures through the C18-filled tips, which were then further washed with 6 × 200 µl of acetonitrile with 0.1% formic acid. For each reaction mixture, all eluted material was combined, and the resulting solution dried under vacuum, then stored at −80 °C. Each reaction mixture was dissolved in 100 µl of 50% acetonitrile, 50% water and 0.1% formic acid. 80 µl of this mixture was injected onto a hydro C18 semi-preparative column and eluted using HPLC gradient method 2 using 0.1% formic acid. Six fractions were collected: F1 (0–3.06 min), F2 (3.06–6.05 min), F3 (6.05–9.05 min), F4 (9.05–12.05 min), F5 (12.05–15.05 min) and F6 (15.05–18.05 min). Fractions 3 and 4 were pooled and all fractions were dried by vacuum. Fractions 3, 4 and 5 were analysed by Orbitrap LC-MS (supplementary methods). SIEVE software^70^, was used to analyse LC-MS data, identifying labeled LRC by searching for co-eluting perfect pairs with expected mass differences (Supplementary Table S5).

### LRC characterisation

A reaction mixture containing lysate (made from 30 light organs, 0.0231 g of tissue), unlabeled Tyr, XA and ATP-Mg^2+^ was set up at 10 °C and monitored for luminescence, with further Tyr, XA and ATP-Mg^2+^ added until no further luminescence was produced. Incubation continued for three days, after which LRC was isolated by reverse phase bench column chromatography and HPLC. Isolated LRC was dissolved in 200 µl of 50% acetonitrile, 50% water and 0.1% formic acid, then analysed by HPLC-UV and LC-MS. Initial analysis by Orbitrap LC-MS showed the sample to contain about 10 µg LRC (99% pure). UV-Vis spectra were taken using the HPLC photodiode array. The sample was dried and re-dissolved in 200 µl of 50% d3-acetonitrile and 50% deuterium oxide (D_2_O) and placed into a D_2_O Shigimi tube for analysis on a 500 MHz Varian NMR spectrophotometer using ^1^H Presat (D_2_O) settings for 12 h at 25 °C. Tyr (0.2 mg/ml), and XA (0.2 mg/ml) were analysed under the same conditions. Chemical shifts were referenced to the residual solvent signal for acetonitrile at 1.94 ppm. These solutions were then injected without a column into the Orbitrap MS so the number of exchangeable protons could be studied. The LRC solution was reanalysed by nanoflow orbitrap LC-MS to determine the amount of LRC degradation, then assayed for bioluminescent activity.

## Electronic supplementary material

Supplementary Information 1

Supplementary Information 2
