# Supplementary material for: New Zealand glowworm (Arachnocampa luminosa) bioluminescence is produced by a firefly-like luciferase but an entirely new luciferin
Source: Sci Rep. 2018 Feb 19;8:3278. doi: 10.1038/s41598-018-21298-w (PMC5818473; doi:10.1038/s41598-018-21298-w)
Supplement: Supplementary file 1 — Supplementary Information 1 [file 41598_2018_21298_MOESM1_ESM.pdf]

Supporting Information 1

New Zealand glowworm (*Arachnocampa luminosa*) bioluminescence is produced by a firefly-like luciferase but an entirely new luciferin

Oliver C Watkins[1,2,*], Miriam L Sharpe[1,*], Nigel B Perry[2,**], and Kurt L Krause[1,**]

[1]Department of Biochemistry, University of Otago, Dunedin, New Zealand

[2]New Zealand Institute for Plant and Food Research Ltd., Department of Chemistry, University of Otago, Dunedin, New Zealand

*These authors contributed equally to this work

**Corresponding authors

Figures S1 to S10

Tables S1, S4 to S7

[Tables S2 and S3 are in Supporting information 2 (.xlsx file)]

Fig. S1. Reducing agents extend luminescent activity. Lysate was prepared by homogenising *A. luminosa* light organs in Tricine pH 7.4 buffer, then divided into three equal volumes, all kept at 4°C. Either Dithiothreitol (DTT) or 2-Mercaptoethanol (βME) to a final concentration of 2 mM, or buffer were added to the samples. Subsamples were removed from the samples at various time points over a period of nearly four hours after the lysate was first prepared, and assayed for luminescent activity in 0.1 M Tris pH 8.0 buffer with the addition of ATP-Mg^{2+}.

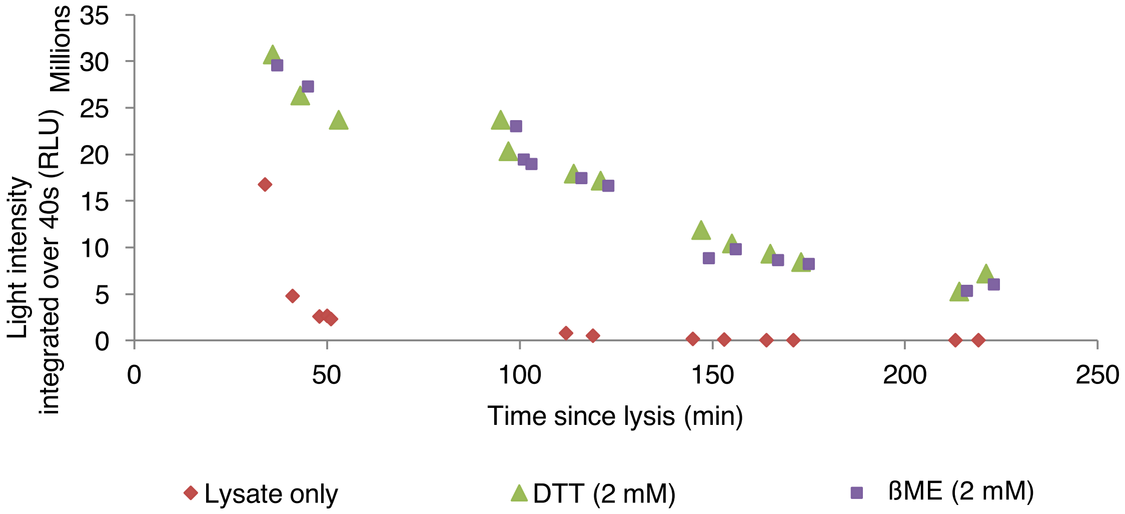

Fig. S2. Screen for optimal buffer and pH in glowworm bioluminescence assay. Previously, glowworm light production was assayed using Tris buffer at pH 8.0 (Viviani *et al.*, 2002). This screen shows that the glowworm system appears to have a preference for pH ranging from 7.0 to 8.0, particularly for MOPS pH 7.5. Lysate prepared from *A. luminosa* light organs was assayed using a range of assay buffer conditions based on a previously published Thermofluor Assay Screen (Laws *et al.*, 1986). Each assay had a final volume of 100 µl: 64 µl of buffer at 0.1 M, 1 µl DTT 200 mM, 5 µl of lysate and 30 µl of ATP-Mg^{2+}. Assays were carried out in duplicate, with a Tris pH 8.0 control assay carried out every sixth assay. Results for each condition in RLU integrated over 40s were averaged and normalized to the results for the next closest control assay.

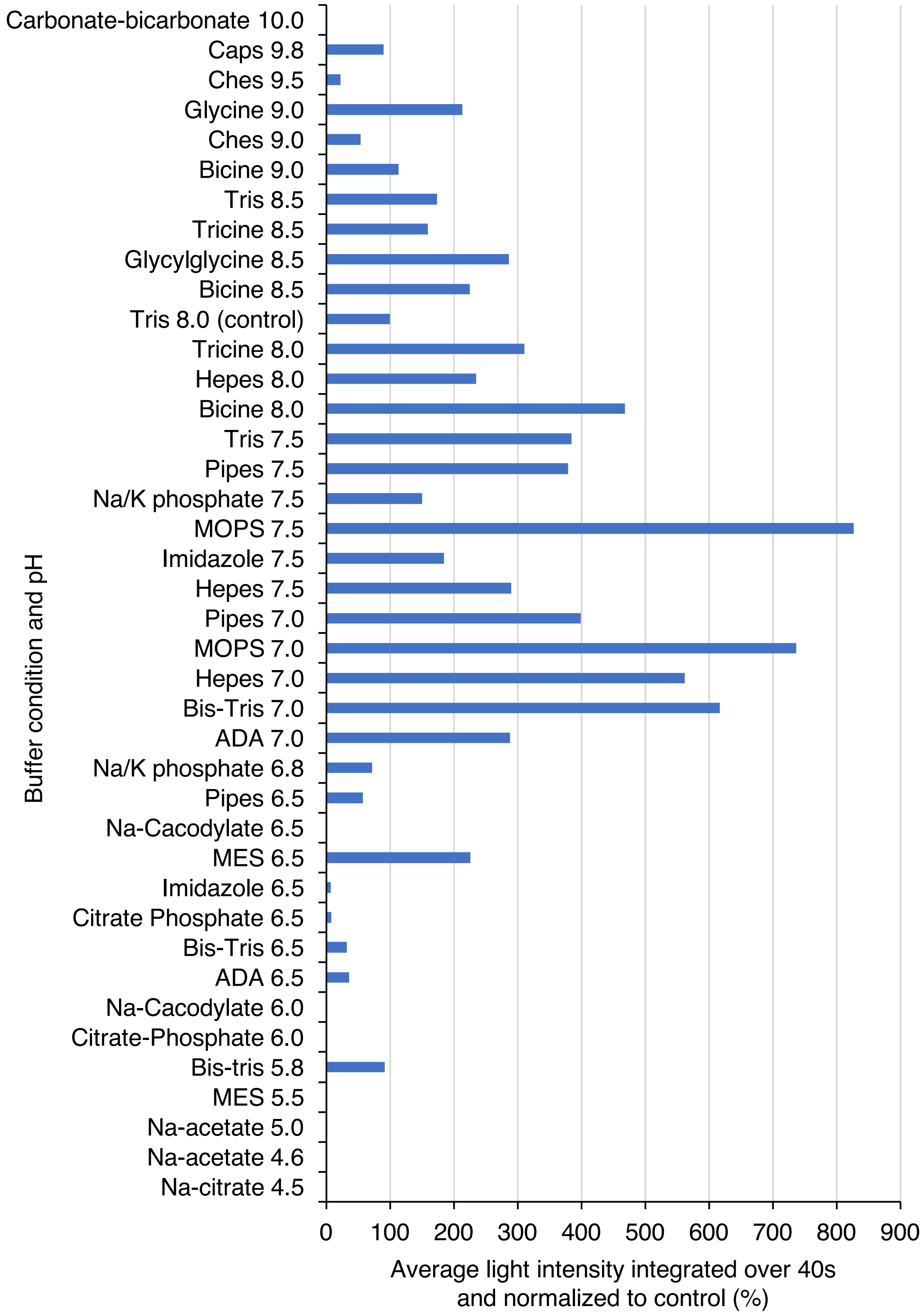

Fig. S3. Addition of non-luminescent tissue to light organ lysate decreases luminescence. Lysates were prepared from either light organs or non-luminescent tissue (glowworm with light organ removed) in Tricine buffer pH 7.4 with 2 mM DTT. Light organ lysate (5 µl) was assayed alone or with non-luminescent tissue lysate (5 µl) added, in Tris buffer pH 8.0 and DTT, with ATP-Mg^{2+} added (final volume of 100 µl). Light organ lysate without non-luminescent tissue added produces more than twice the luminescence than with non-luminescent tissue present (using a student T test, two tailed, two sample unequal variance, showed significance at $P < 0.00005$). Data shows average of three replicate reaction mixtures with error bars showing one standard deviation above and below the mean.

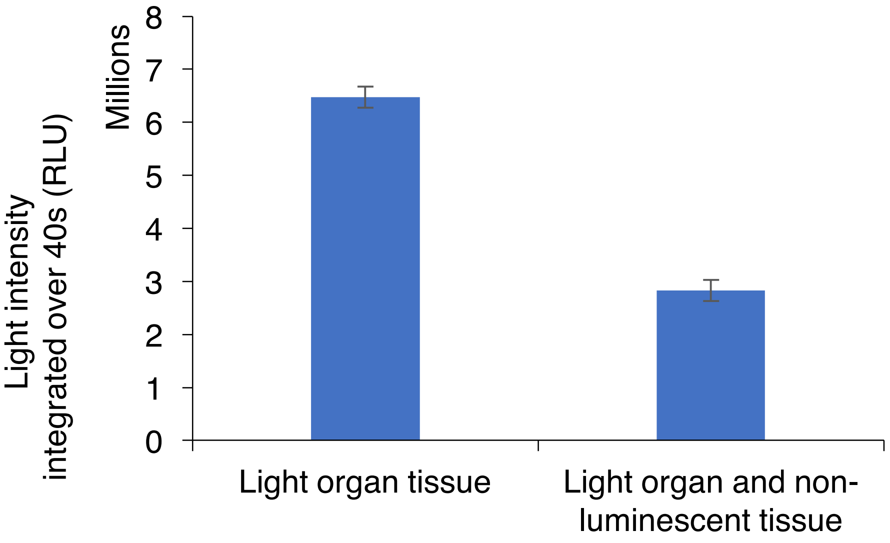

Fig. S4. *A. luminosa* secondary metabolites observed in light organ lysate by direct injection Orbitrap mass spectrometry in positive (A) and negative (B, C) ion modes. These spectra show that firefly luciferin was not a component of glowworm light organs. The characteristic [M-H]$^-$ and decarboxylated daughter ion of firefly D-luciferin have the compositions $C_{11}H_7N_2O_3S_2^-$ (278.9895) and $C_{10}H_7N_2OS_2^-$ (234.9996) (22). Lysate was made from 14 light organs homogenised in 1:1 water:acetonitrile; mass spectrometry taken 5 to 6 min after lysis.

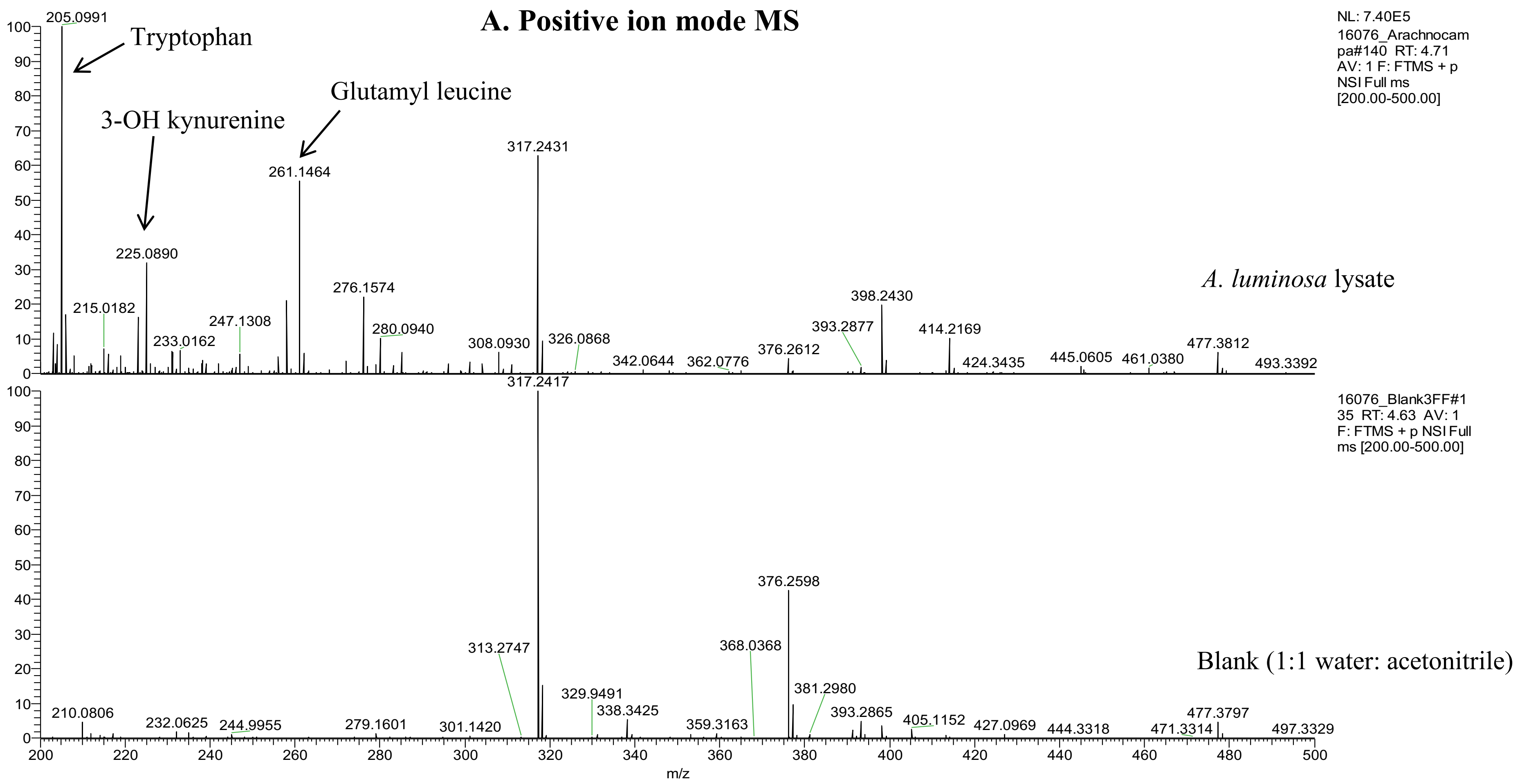

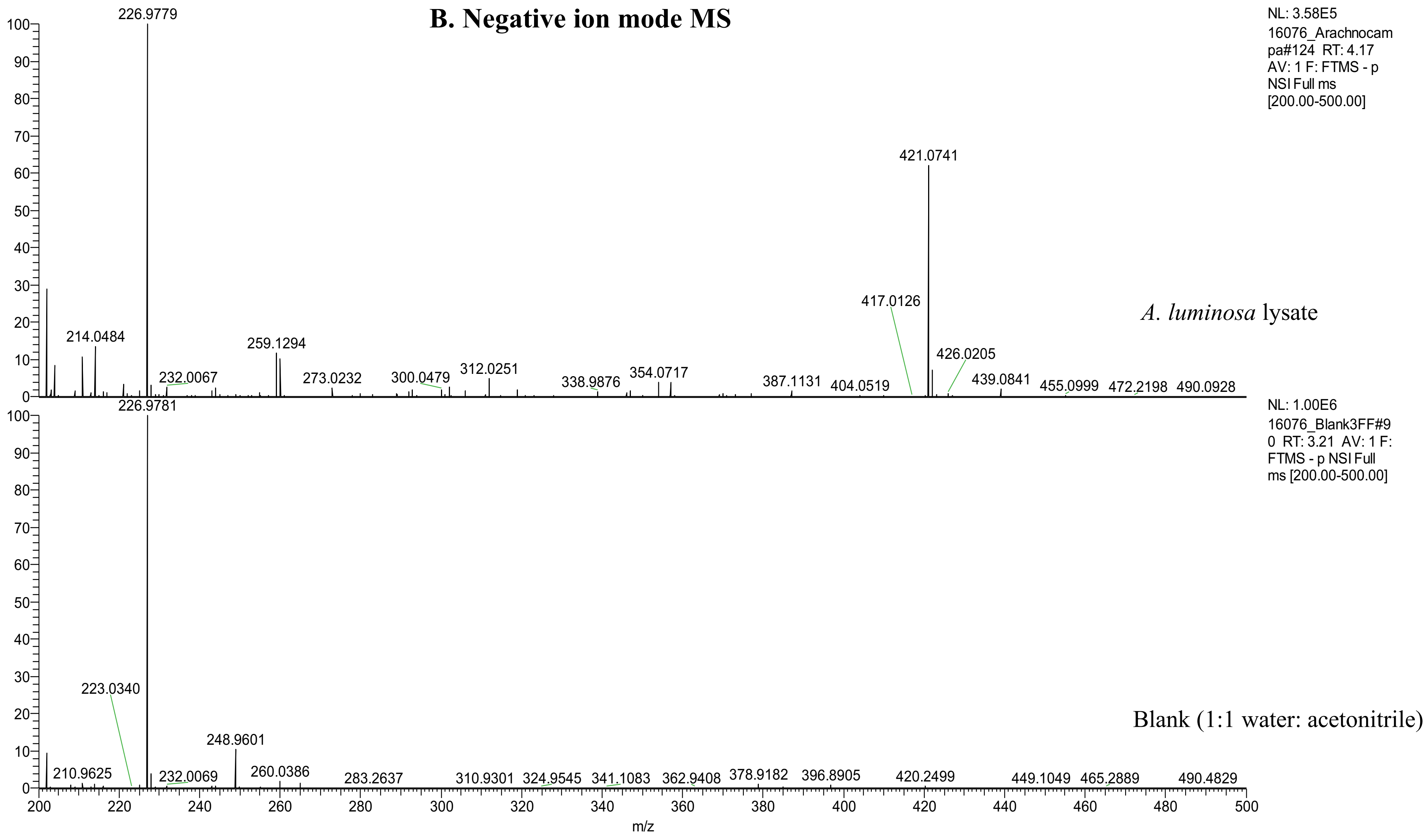
B. Negative ion mode MS
NL: 3.58E5
16076_Arachnocampa#124 RT: 4.17
AV: 1 F: FTMS - p NSI Full ms [200.00-500.00]
226.9779
421.0741
417.0126
214.0484
259.1294
426.0205
232.0067
273.0232
300.0479
312.0251
338.9876
354.0717
387.1131
404.0519
439.0841
455.0999
472.2198
490.0928
A. luminosa lysate
NL: 1.00E6
16076_Blank3FF#90 RT: 3.21 AV: 1 F: FTMS - p NSI Full ms [200.00-500.00]
226.9781
223.0340
248.9601
210.9625
232.0069
260.0386
283.2637
310.9301
324.9545
341.1083
362.9408
378.9182
396.8905
420.2499
449.1049
465.2889
490.4829
Blank (1:1 water: acetonitrile)
m/z

C. Expansion of negative ion mode MS

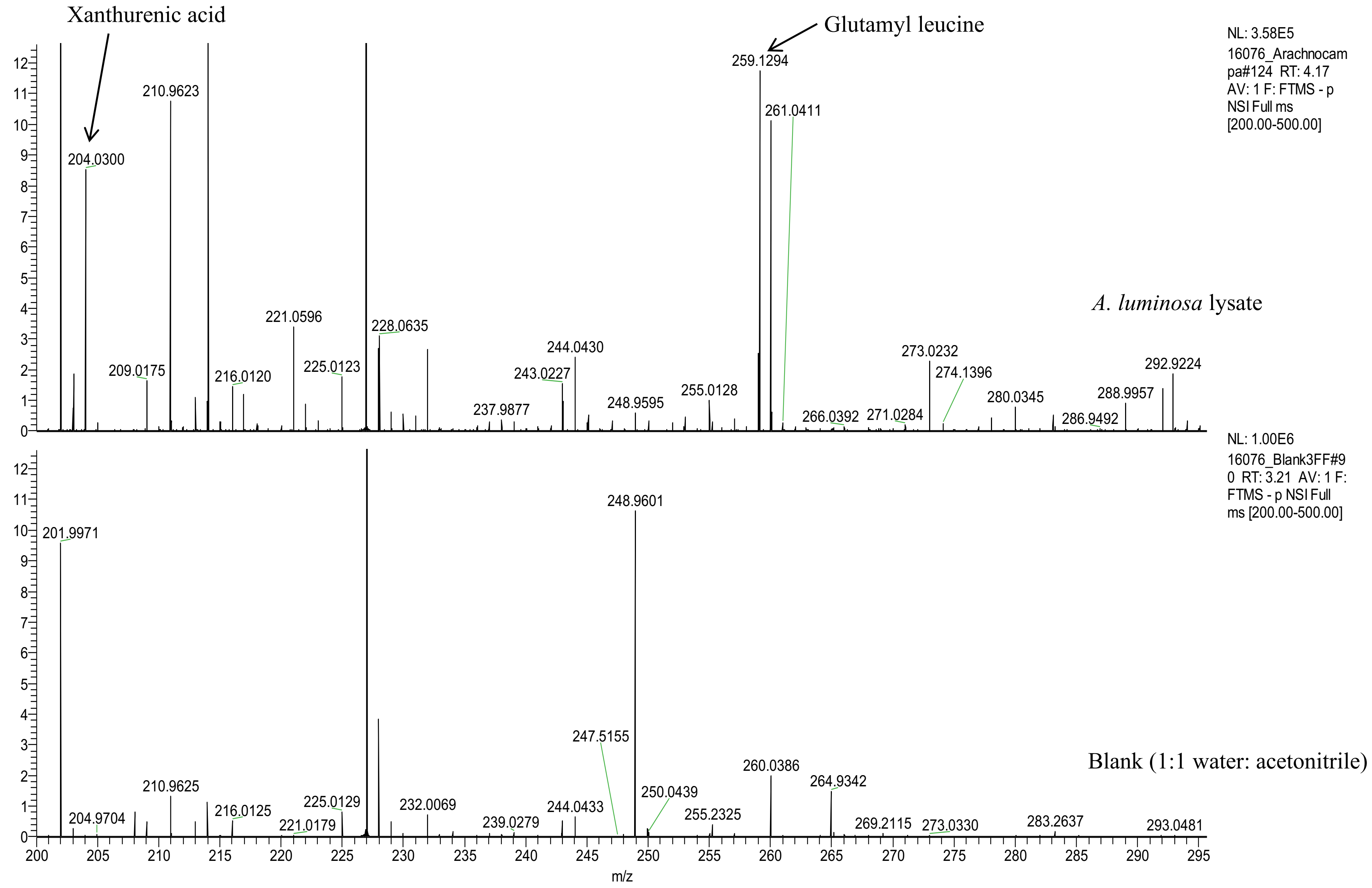

Fig. S5. Alignment of the four larval serum protein 2 sequences identified in the bioluminescent active fraction purified from glowworm light organ. Residues are colored according to conservation of sequence identity (dark blue: 100 % conserved). The alignment was carried out using CLC Genomics Workbench (version 8.5.1; http://www.clcbio.com) and visualised using Jalview (http://www.jalview.org).

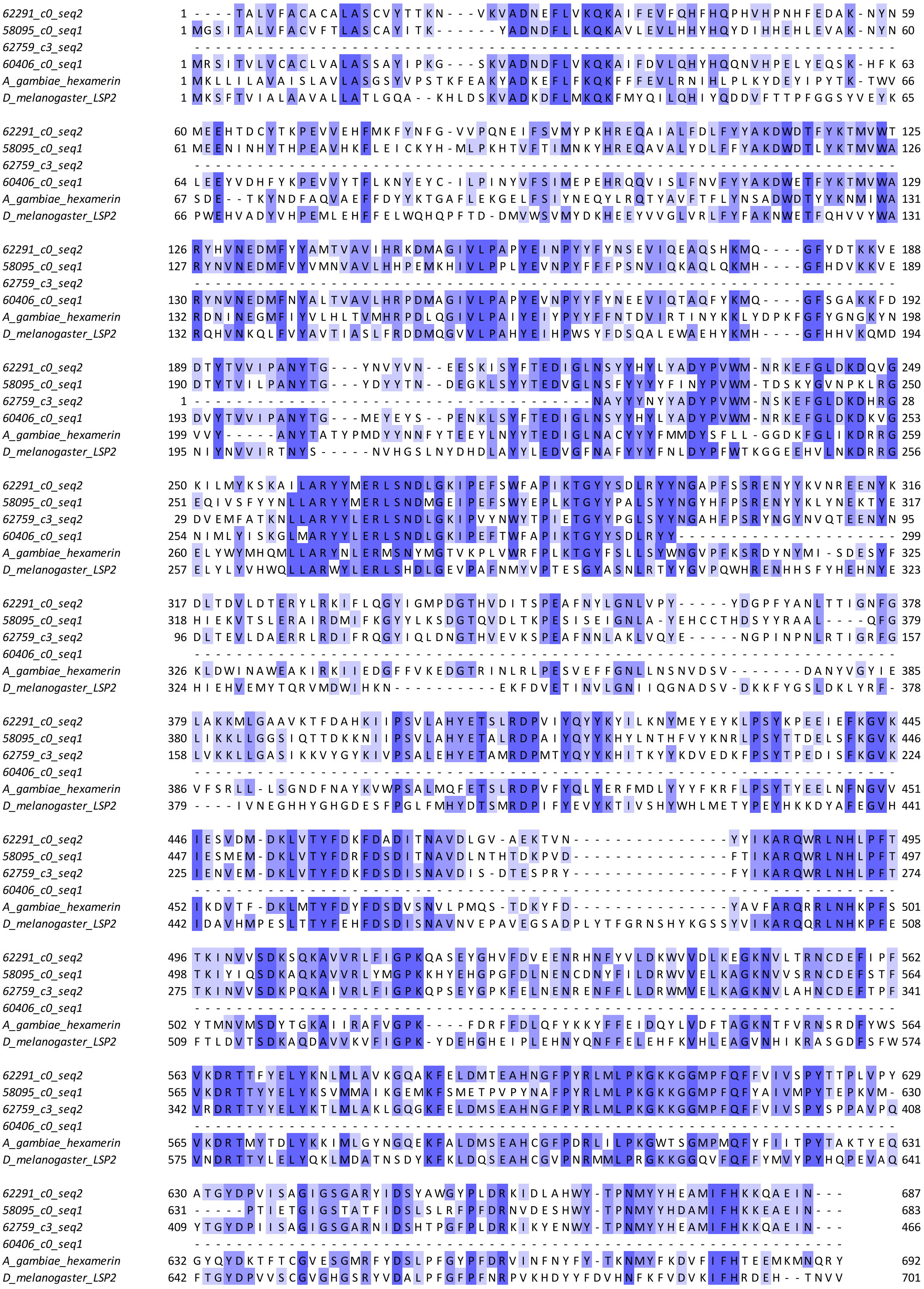

Fig. S6. Alignment of the two luciferin 4-monooxygenase sequences identified in the bioluminescent active fraction purified from glowworm light organ. Residues are colored according to conservation of sequence identity (dark blue: 100 % conserved). The alignment was carried out using CLC Genomics Workbench (version 8.5.1; http://www.clcbio.com) and visualised using Jalview (http://www.jalview.org).

```
55693_c1_seq1     1 MAKVVDNIVYGAPPEFDVLKEANSYGEYLFKRLKARGDEVSVVDGLTGEQ 50
62762_c0_seq1     1 MAKVVDNIVYGAPPEFDVLKEANSYGEYIFKRLKARGDEVSVVDGLTGEQ 50

55693_c1_seq1    51 IRASDIYSKVVRTAECLQAYGIKKGDRVGICSDTMIEYYYIVMGTMAVGA 100
62762_c0_seq1    51 VRASDIYSKVVRTAECLQAYGIKKGDRVGICSDTMIEYYYIVMGTMAVGA 100

55693_c1_seq1   101 IICPVIISWTESDMNHAFNLSCPTIFFVSKIILERIAKLAKRNPYVKDII 150
62762_c0_seq1   101 IICPVIISWTESDMNHAFNLSCPTIFFVSKIILERIAKLAKRNPYVKDII 150

55693_c1_seq1   151 VFDDDAPEKPFISFKDFLANPKIPSRPHFDCQPQDMETNVCAVLLTSGTT 200
62762_c0_seq1   151 VFDDDAPEKPFISFKDFLANPKIPSRPHFDCQPQDMETNVCAVLLTSGTT 200

55693_c1_seq1   201 GLSKGVAISQYNLIHFMSLDTKSDKRGLFLCVAQYSNAFGFTALMRRTFN 250
62762_c0_seq1   201 GLSKGVAISQYNLIHFMSLDTKSDKRGLFLCVAQYSNAFGFTALMRRTFN 250

55693_c1_seq1   251 GTRVIHLPR----------------------------------------- 259
62762_c0_seq1   251 GTRVIHLPRYEEKAYLECVQKYKVNYISVHPPLMLSLAKKPEIDNYDLSS 300

55693_c1_seq1       --------------------------------------------------
62762_c0_seq1   301 IERIYCSGTTVSVRILYAVAERLGVNYVRQFYGSSECLAVVAQSNEYSTK 350

55693_c1_seq1       --------------------------------------------------
62762_c0_seq1   351 GSVGRLMPGIIGKVVHTETGAHLPANERGYLKFKANSTMYGYYNNPEASK 400

55693_c1_seq1       --------------------------------------------------
62762_c0_seq1   401 VVKDEEGYVNTGDVGYYNERLEWFVVDRLKDIVMVEGVPVAPTEMETTLL 450

55693_c1_seq1       --------------------------------------------------
62762_c0_seq1   451 LHPDIIDACVIGISDGKGGEVLFAFLTKTRDVTEKDVMAFVAEKMPYPKH 500

55693_c1_seq1       ----------------------------
62762_c0_seq1   501 LSGGCQFVEEIPKNPAGKMLRRILRGTL                       528
```

Fig. S7. Alignment of luciferase sequences from glowworms *A. luminosa* and *A. richardsae*, and fireflies *P. pyralis* and *L. cruciata*. Residues are colored according to conservation of sequence identity (dark blue: 100 % conserved). Black boxes represent positions of ATP-binding motifs conserved throughout the ANL superfamily (13), and red boxes represent luciferin-binding residues from the beetle luciferase (Branchini *et al.*, 2001; Branchini *et al.*, 2003). The residue marked with a '#' plays a key role in the firefly luciferase adenylation half reaction, and the residue marked with a '*' plays a key role in the oxidation (light-producing) half reaction (Branchini *et al.*, 2005). The alignment was carried out using CLC Genomics Workbench (version 8.5.1; http://www.clcbio.com) and visualised using Jalview (http://www.jalview.org).

```
P_pyralis       1 MEDAKN---IKKGPAPFYPL--EDGTAGEQLHKAMKRYALVPGTIAFTDAHIEVNITY 53
L_cruciata      1 MENMENDENIVVGPKPFYPI--EEGSAGTQLRKYMERYAKL-GAIAFTNAVTGVDYSY 55
A_luminosa      1 MAKV-VD-NIVYGAPPEFDVLKEANSYGEYIFKRLKARG---DEVSVVDGLTGEQVRA 53
A_richardsae    1 MACTSVN-NIVYGPKPTFDVLKEANSYGEYAFKRLRARG---DEVSVIDALTGEEIRA 54

P_pyralis      54 AEYFEMSVRLAEAMKRYGLNTNHRIVVCSENSLQFFMPVLGALFIGVAVAPANDIYNE 111
L_cruciata     56 AEYLEKSCCLGKALQNYGLVVDGRIALCSENCEEFFIPVIAGLFIGVGVAPTNEIYTL 113
A_luminosa     54 SDIYSKVVRTAECLQAYGIKKGDRVGICSDTMIEYYYIVMGTMAVGAIICPVIISWTE 111
A_richardsae   55 SDIYAKTVRTAECLQAYGIRKGDRVGICSDTMIEYYYIVMGTMAVGAIICPIIISWTE 112

P_pyralis     112 RELLNSMNISQPTVVFVSKKGLQKILNVQKKLPIIQKIIIMDSKTDYQGFQSMYTFVT 169
L_cruciata    114 RELVHSLGISKPTIVFSSKKGLDKVITVQKTVTTIKTIVILDSKVDYRGYQCLDTFIK 171
A_luminosa    112 SDMNHAFNLSCPTIFFVSKIILERIAKLAKRNPYVKDIIVFDDDAPEKPFISFKDFLA 169
A_richardsae  113 ADMNHAFNISCPTVFFVSKSILPTIARIAKRNPYVKDIIVFDDNAPEKPLISFKDFLA 170

P_pyralis     170 SHLPPGFNEYDFVPESFDRDKTIALIMNSSGSTGLPKGVALPHRTACVRFSHARDPIF 227
L_cruciata    172 RNTPPGFQASSFKTVEVDRKEQVALIMNSSGSTGLPKGVQLTHENTVTRFSHARDPIY 229
A_luminosa    170 NPKIPSRPHFDCQPQ--DMETNVCAVLLTSGTTGLSKGVAISQYNLIHFMS------L 219
A_richardsae  171 NPKVPSKPHFDCEPQ--DMENTIATVLLTSGTTGISKGVAISQYNLIHFMS------L 220

P_pyralis     228 GNQIIPDTAILSVVPFHHGFGMFTTLGYLICGFRVVLMYRFEEELFLRSLQDYKIQSA 285
L_cruciata    230 GNQVSPGTAVLTVVPFHHGFGMFTTLGYLICGFRVVMLTKFDEETFLKTLQDYKCTSV 287
A_luminosa    220 DTKSDKRGLFLCVAQYSNAFGFTALMRRTFNGTRVIHLPRYEEKAYLECVQKYKVNYI 277
A_richardsae  221 DTKTYKKGLFLCVAQYSNAFGFTALMRRAFNGTRVLHLPRYDEKSYLECVQKFKVNYI 278

P_pyralis     286 LLVPTLFSFFAKSTLIDKYDLSNLHEIASGGAPLSKEVGEAVAKRFHLPGIRQGYGLT 343
L_cruciata    288 ILVPTLFAILNKSELLNKYDLSNLVEIASGGAPLSKEVGEAVARRFNLPGVRQGYGLT 345
A_luminosa    278 SVHPPLMLSLAKKPEIDNYDLSSIERIYCSGTTVSVRILYAVAERLGVNYVRQFYGSS 335
A_richardsae  279 SVHPPLMLSLAKKPEIANYDLSSLERIYCSGTTVSVRILYQVAERIGVKVVRQFYGSS 336

P_pyralis     344 ETTSAILITPEGDDKPGAVGKVVPFFEAKVVDLDTGKTLGVNQRGELCVRGPMIMSGY 401
L_cruciata    346 ETTSAIIITPEGDDKPGASGKVVPLFKAKVIDLDTKKSLGPNRRGEVCVKGPMLMKGY 403
A_luminosa    336 ECLAVVAQSNEYSTK-GSVGRLMPGIIGKVVHTETGAHLPANERGYLKFKANSTMYGY 392
A_richardsae  337 ECLAVVAQSDEFCTK-GSVGTLMPGIIGKVIHPETGALLGPNERGFLKFKANSTMYGY 393

P_pyralis     402 VNNPEATNALIDKDGWLHSGDIAYWDEDEHFFIVDRLKSLIKYKGYQVAPAELESILL 459
L_cruciata    404 VNNPEATKELIDEEGWLHTGDIGYYDEEKHFFIVDRLKSLIKYKGYQVPPAELESVLL 461
A_luminosa    393 YNNPEASKVVKDEEGYVNTGDVGYYNERLEWFVVDRLKDIVMVEGVPVAPTEMETTLL 450
A_richardsae  394 FNNPEASKVVKDEEGYVNTGDAGYYNERFEWFVVDRLKDIVMVDGVAVAPTEMETTIL 451
                                                           *

P_pyralis     460 QHPNIFDAGVAGLPDDDAGELPAAVVVLEHGKTMTEKEIVDYVASQVTTAKKLRGGVV 517
L_cruciata    462 QHPSIFDAGVAGVPDPVAGELPGAVVVLESGKNMTEKEVMDYVASQVSNAKRLRGGVR 519
A_luminosa    451 LHPDIIDACVIGISDGKGGEVLFAFLT-KTR-DVTEKDVMAFVAEKMPYPKHLSGGCQ 506
A_richardsae  452 LHPDIIDACVIGISDGEGGEVLFAFLT-KTRKEVTEKDVMDFVAEKLPYPKHLKGGCQ 508

P_pyralis     518 FVDEVPKGLTGKLDARKIREILIKAKKGGKSKL                          550
L_cruciata    520 FVDEVPKGLTGKIDGRAIREILKKPV----AKM                          548
A_luminosa    507 FVEEIPKNPAGKM----LRRIL-------RGTL                          528
A_richardsae  509 FVDEIPKNPAGKM----LRRIL-------RGTL                          530
                             #
```

Fig. S8. LC-MS analysis of HPLC active fractions from separations of hot extract. (A) Ion Trap LC-MS in negative mode of HPLC active fraction from separation 2. (B) Orbitrap LC-MS in positive mode of HPLC active fraction from separation 3.

A

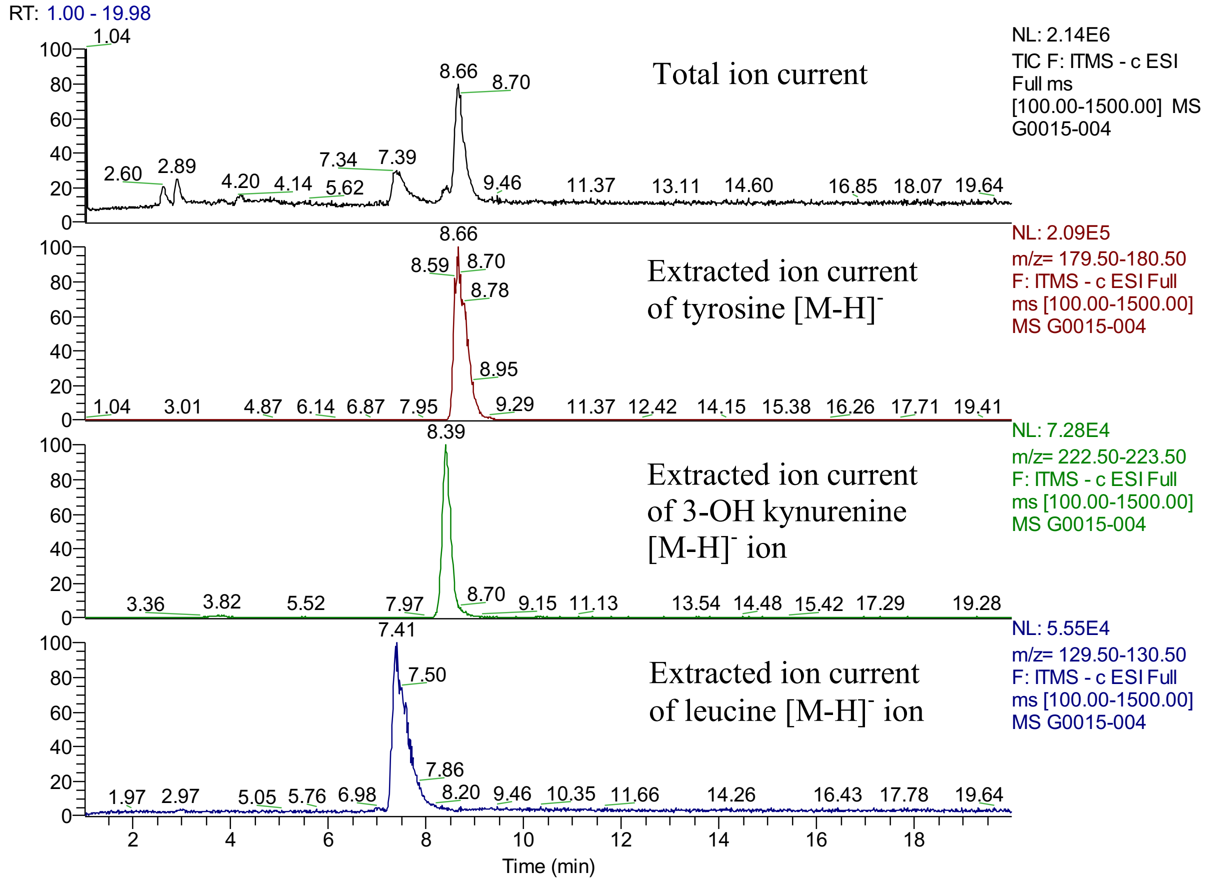

B

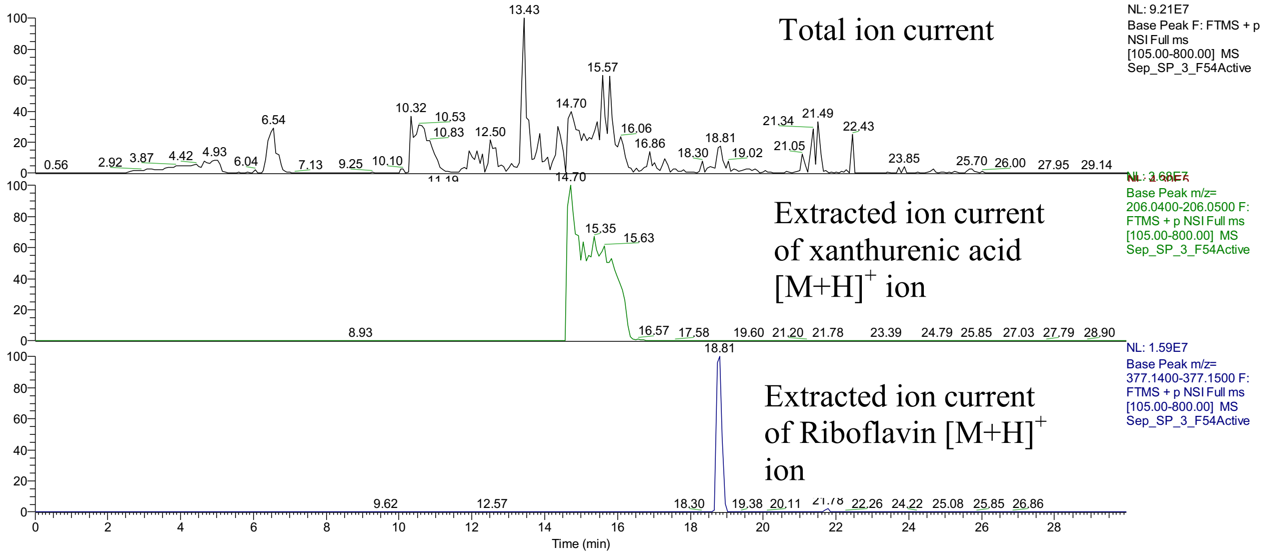

Fig. S9. MS of LRC from reaction mixtures containing crude luciferase, labeled (A) or unlabeled (B) tyrosine, xanthurenic acid and ATP-Mg^{2+}). Orbitrap nanoflow LC-MS of unlabeled LRC at m/z 369 Da (A), and labeled LRC at m/z 379 Da (B). MS^2 fragmentation of LRC ions 369 Da (C) and 379 Da (D). Higher-energy collisional dissociation (HCD) fragmentation energy: 45 v. m/z 50 – 380 Da region.

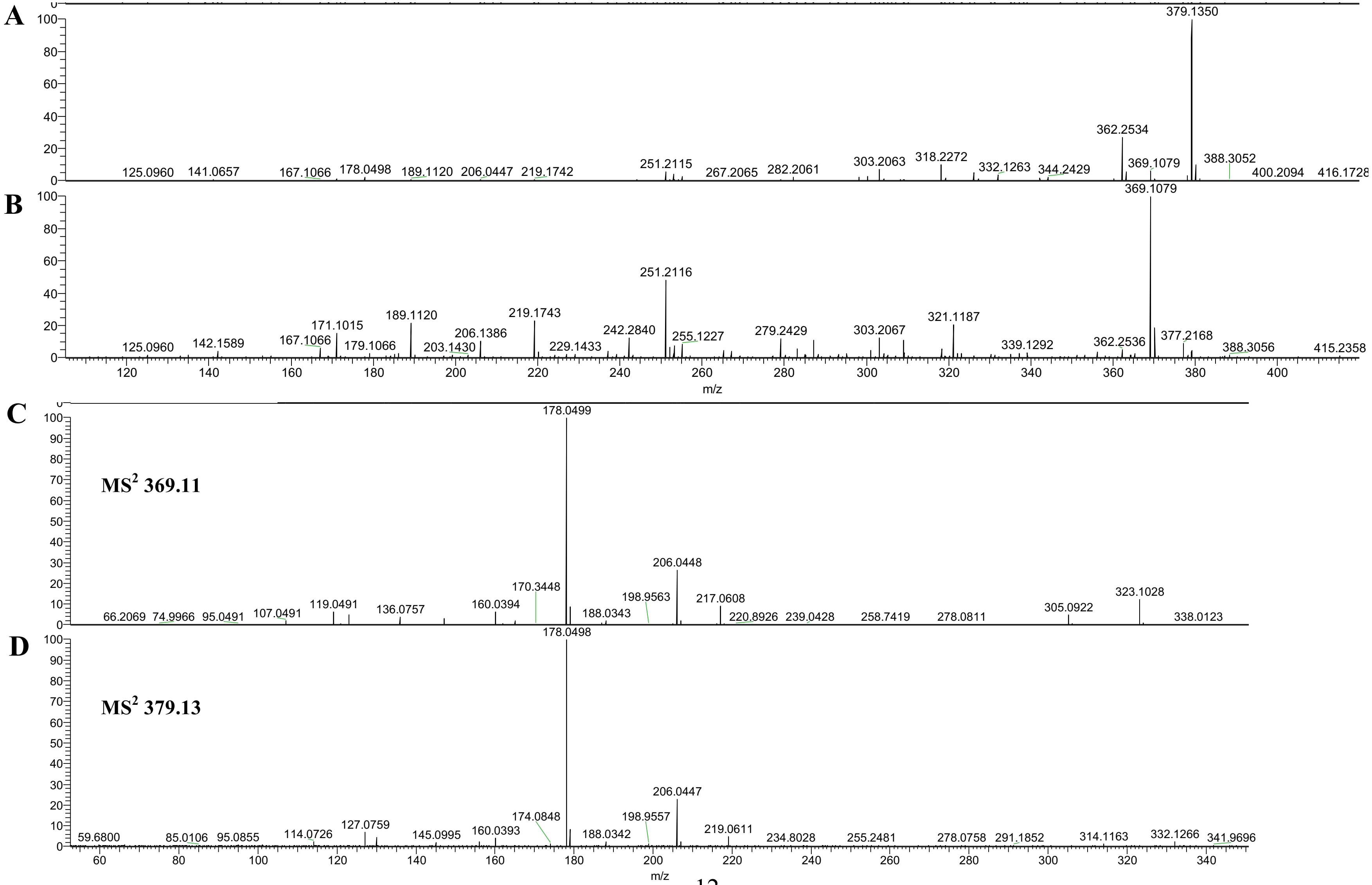

Fig. S10. ¹H NMR spectra of LRC, tyrosine and xanthurenic acid. (A) Full spectra. (B) Expanded spectra (3.5 – 6.0 ppm); HOD peak is larger in the LRC spectrum due to the low concentrations of LRC (50 µg/ml). (C) Expanded spectra (6.9 – 9.3 ppm). The unknown broad peak shown has an integration less than one proton and was not observed when the spectra of LRC was taken a few weeks later after desiccation and re-solubilisation. The unknown broad peak is therefore not thought to be due to the dominant tautomer of LRC and likely represents a minor tautomer of LRC or solvent contamination. LRC (red), tyrosine (Tyr, green) and xanthurenic acid (XA, blue) ¹H NMR spectra in D_2O: d_3-MeCN (1:1) (500 MHz referenced to 1.94 ppm MeCN peak). Arrows show peak assignments.

A

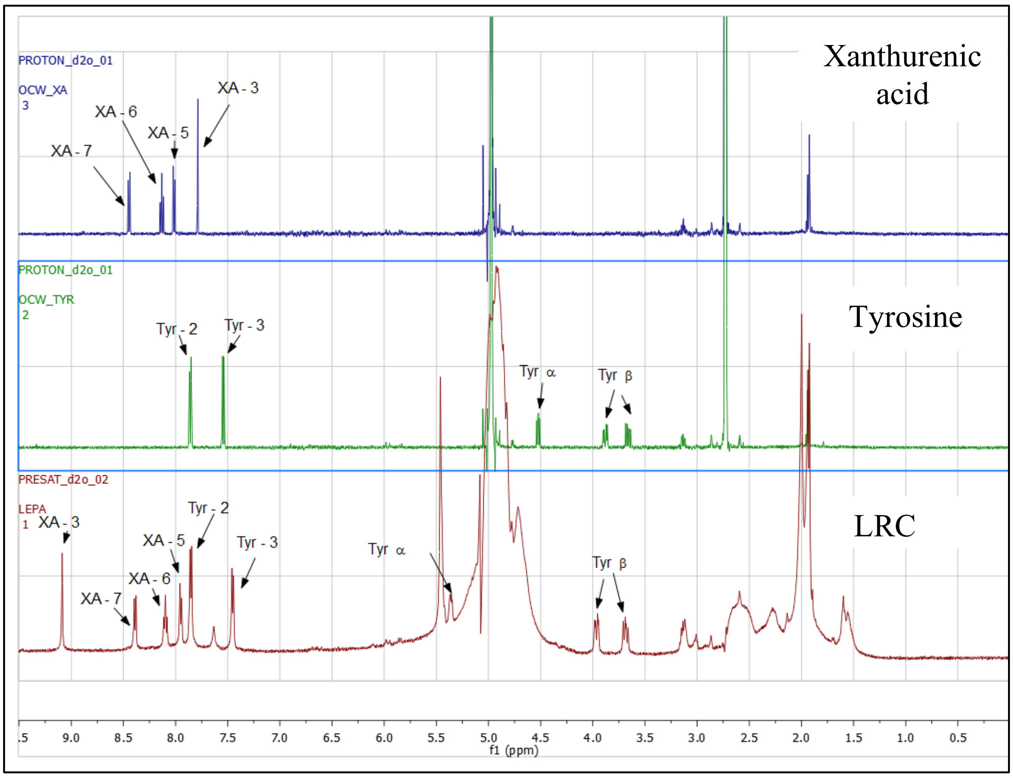

B

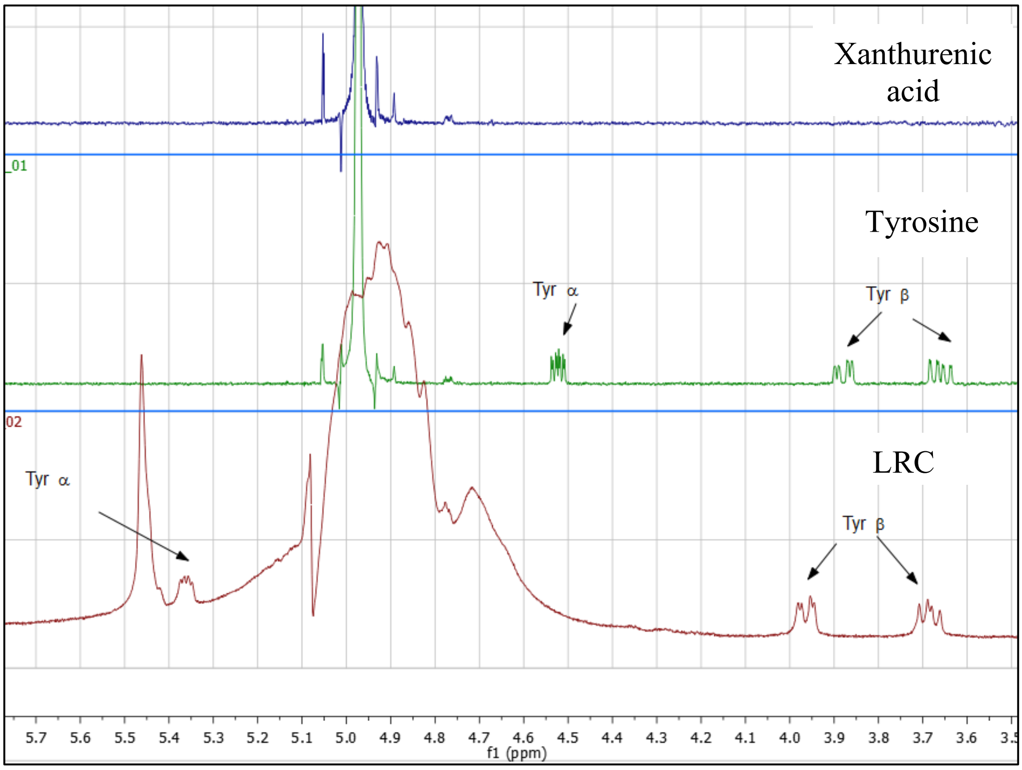

C

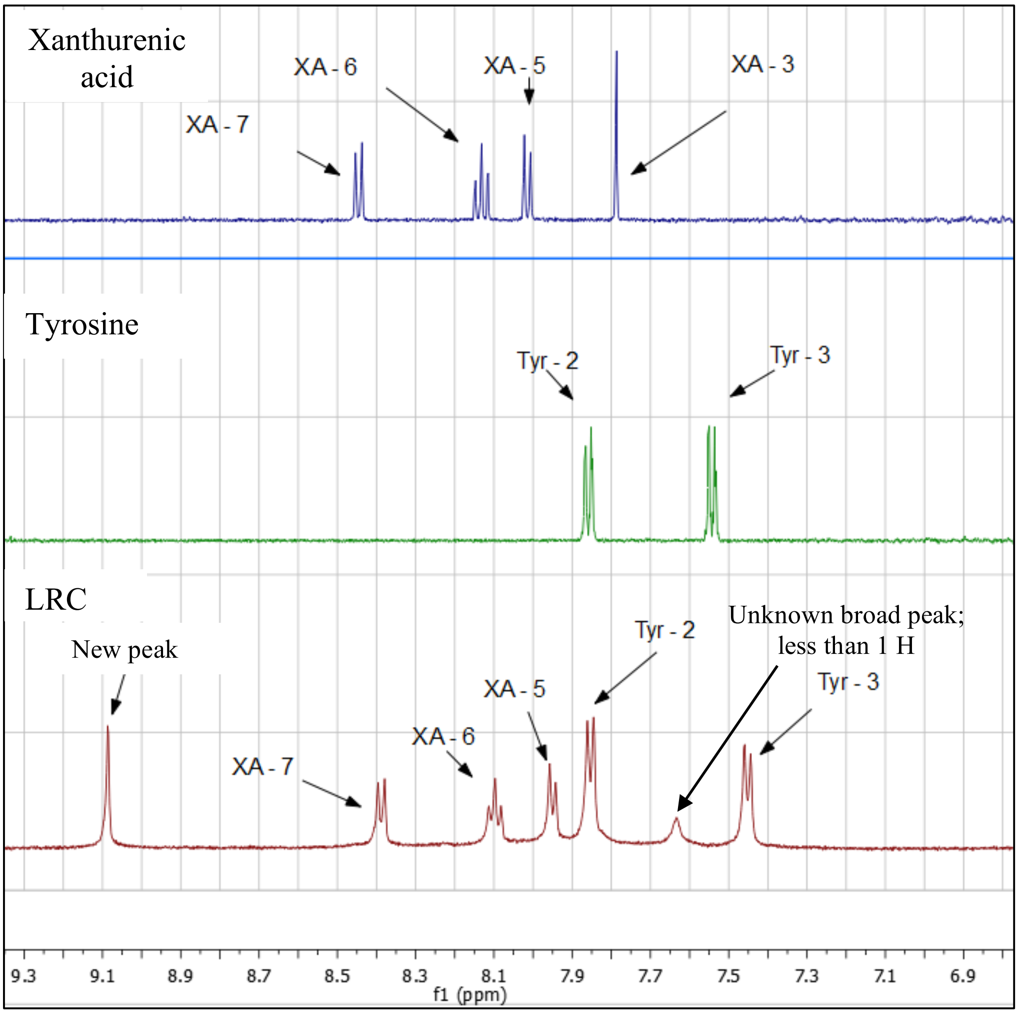

Xanthurenic acid
XA - 7
XA - 6
XA - 5
XA - 3
Tyrosine
Tyr - 2
Tyr - 3
LRC
New peak
XA - 7
XA - 6
XA - 5
Tyr - 2
Unknown broad peak; less than 1 H
Tyr - 3
9.3
9.1
8.9
8.7
8.5
8.3
8.1
7.9
7.7
7.5
7.3
7.1
6.9
f1 (ppm)

Table S1. ***A. luminosa*** **secondary metabolites measured by Orbitrap nanoflow LC-MS.** Concentration (conc, nMol/mg of frozen light organs) of major components found in glowworm lysates and crude luciferase extracts, as estimated by LC-MS nanoflow Orbitrap. Table shows average of three technical replicates. Standard deviation was 20% between replicates for major compounds shown. The variance was not decreased when peak areas were normalized to any of the major compounds (which should act as natural internal standards). * Matches literature MS and MS^2 (Yanshole *et al.*, 2010); ** matches standard MS^2 and retention time.

**Major components**	**Ion $[M+H]^+$**	**Retention time nanoflow**	**Retention time milliflow**	**Concentration (nMol/mg of frozen light organs)**			**Increased (+) or decreased (-) in crude luciferase extract compared to lysate**
				**Lysate 1**	**Lysate 2**	**Crude luciferase extract**	
Leucine**	132.1019	5.0	7.5	1.79	2.00	1.34	-
3-OH kynurenine**	225.0867	6.0	7.8	Trace	Trace	Trace	-
Tyrosine**	182.0811	6.3	10.4	1.11	1.25	0.63	-
Inosine monophosphate**	349.0548	6.5	6	4.31	1.63	0.15	-
Glutathione **(oxidised)	613.1594	6.5	10.2	Trace	Trace	Trace	-
Glutathione (reduced)**	307.0794	9.8	11.0	Trace	Trace	Trace	-
Guanosine**	284.0991	7.5	11.4	1.48	0.59	0.68	-
Inosine**	269.088	7.7	11.5	6.17	2.34	Trace	-
Phenyl alanine**	166.088	7.7	14.1	0.44	0.21	1.30	+
Glutamyl valine*	247.1288	7.9	13.3	4.70	1.87	Trace	-
Glutamyl tyrosine**	311.1237	9.3	18.5	5.46	2.09	Trace	-
Tryptophan**	205.0925	10.3	28.2	0.35	0.15	0.75	+
Hydroxy tryptophan*	221.092	10.3	29	2.82	1.07	Trace	-
Unknown A	237.0869	10.4	29	4.04	1.48	Trace	-
Glutamyl leucine*	261.1444	12.9	30	8.79	3.49	1.41	-
Xanthurenic acid**	206.0404	14.4	33.6	0.61	0.23	1.31	+
Unknown B	261.0869	16.6	30.8	1.13	0.36	0.30	-
Riboflavin**	377.1451	18.0	36	0.99	0.38	0.15	-

Table S4. Summary of sequential chromatographic steps used to obtain luciferin-containing luminescent fractions from A. luminosa light organ hot extract on three different separations. The calculated luminescence yield (LY) compared with the initial hot extract sample at each stage is provided.

	**Separation 1**	**Separation 2**	**Separation 3**
Starting material	Hot extract	Hot extract	Hot extract
Reducing agent used in mobile phase during fractionation	Dithiothreitol	No reducing agent	Tris (2-carboxyethyl) phosphine and β-mercaptoethanol for step 1, β-mercaptoethanol for step 2
Chromatography step 1	*Reverse phase bench chromatography (gradient of water to acetonitrile)*<br>*Eluted fractions concentrated and assayed for presence of luciferin*		
Active fraction selected for step 2	Fraction with FP type time course activity eluted with 20% acetonitrile<br>LY 71%	Fraction with SP type time course activity eluted with water<br>LY 10%	Fraction with SP type time course activity eluted with 20% acetonitrile<br>LY 186%
Chromatography step 2	*Reverse-phase HPLC (gradient of water to acetonitrile)*<br>*Eluted fractions concentrated and assayed for presence of luciferin*		
Final active fraction selected for LC-MS analysis	Fraction with FP type time course activity eluted with 6% acetonitrile at 20 min<br>LY 64%	Fraction with SP type time course activity eluted with 6% acetonitrile at 8 min<br>LY 3%	Fraction with SP type time course activity eluted with 100% acetonitrile at 35 min<br>LY 21%
Identity of major compound in final active fraction (LC-MS)	Not enough material	Tyr	XA

Table S5. MS and MS^2 data for unlabeled and labeled LRC and XA using Orbitrap nanoflow LC-MS, positive ion mode. XA*, ULTyr* LABTYR* indicate ions observed from the fragmentation of XA, unlabled tyrosine and labelled tyrosine respectively.

Unlabeled LRC			Labeled LRC		
**Ion Da (relative intensity)**	**Assignment**	**Calculated Da**	**Ion Da (relative intensity)**	**Assignment**	**Calculated Da**
369.108	$[M+H]^+$ $C_{19}H_{17}N_2O_6$	369.108	379.135	$[M+H]^+$ $C_{10}{}^{13}C_9H_{17}N^{15}NO_6$	379.135
**MS^{2}of 369.108**			**MS^{2}of 379.135**		
323.103 (12)	$[M\text{-}CO_2H_2]^+$ $C_{18}H_{15}N_2O_4$	323.103	332.126 (4)	$[M\text{-}{}^{13}CO_2H_2]^+$ $C_{10}{}^{13}C_8H_{15}N^{15}NO_4$	332.126
305.092 (6)	$[M\text{-}CO_2H_2\text{-}H_2O]^+$ $C_{18}H_{13}N_2O_3$	305.092	314.116 (1)	$[M\text{-}{}^{13}CO_2H_2\text{-}H_2O]^+$ $C_{10}{}^{13}C_8H_{13}N^{15}NO_3$	314.116
217.060(10)	$[XA\text{-}O+CNH_4]^+$ $C_{11}H_9N_2O_3$	217.061	219.061 (6)	$[XA\text{-}O+{}^{13}C^{15}NH_4]^+$ $C_{10}{}^{13}CH_9N^{15}NO_3$	219.062
206.045 (28)^{XA*}	$[XA+H]^+$ $C_{10}H_8NO_4{}^+$	206.045	206.045 (14)^{XA*}	$[XA+H]^+$ $C_{10}H_8NO_4$	206.045
178.050 (100)^{XA*}	$[XA+H–CO]^+$ $C_9H_8NO_3$	178.050	178.050 (100)^{XA*}	$[XA+H–CO]^+$ $C_9H_8NO_3$	178.050
160.039 (6)^{XA*}	$[XA+H–CO_2H_2]^+$ $C_9H_6NO_2$	160.040	160.039 (6)^{XA*}	$[XA+H–CO_2H_2]^+$ $C_9H_6NO_2$	160.040
136.076 (4)^{ULTyr*}	$[Tyr+H\text{-}CO_2H_2]^+$ $C_8H_{10}NO$	136.076	145.099 (2)^{LABTyr*}	[labeled Tyr+H-$^{13}CO_2H_2]^+$ $^{13}C_8H_{10}{}^{15}NO$	145.100
119.049 (6)^{ULTyr*}	Tyr fragment C_8H_7O	119.049	127.076 (6)^{LABTyr*}	labeled Tyr fragment $^{13}C_8H_7O$	127.075

XA		
**Ion Da (relative intensity)**	**Assignment**	**Calculated Da**
206.045	$[XA+H]^+$ $C_{10}H_8NO_4{}^+$	206.0450
**MS^{2}of 206.045**		
188.0342 (3)	$[XA+H–H_2O]^+$ $C_{10}H_6NO_3$	188.0348
178.050 (50)	$[XA+H–CO]^+$ $C_9H_8NO_3$	178.050
160.039 (3)	$[XA+H–CO_2H_2]^+$ $C_9H_6NO_2$	160.040

Table S6. ^{1}H NMR spectra of LRC, Tyr and XA in 1:1 D_2O:CD_3CN. Shift on ppm (multiplicity, coupling, integration). Tyr assignments from Laws *et al.* (1986), XA assignments from Yanshole *et al.* (2010).

LRC	Tyr	XA	Assignment
9.09 (s, 1H)	-	-	LRC new peak
8.39 (d, 8.3 Hz, 1H)	-	8.45 (d, 8.4 Hz, 1H),	XA 7
8.10 (t, 8.0 Hz, 1H)	-	8.13 (t, 8.1 Hz, 1H),	XA 6
7.95 (d, 7.7 Hz, 1H)	-	8.01 (d, 7.8 Hz, 1H),	XA 5
-	-	7.79 (s, 1H).	XA 3
7.85 (d, 8.0 Hz, 2H)	7.86 (d, 8.5 Hz, 2H)	-	Tyr 2+6
7.45 (d, 8.1 Hz, 2H)	7.54 (d, 8.5 Hz, 2H)	-	Tyr 3+5
5.36 (dd, 9.2, 4.6 Hz, 1H)	4.52 (dd, 8.4, 4.8 Hz, 1H)	-	Tyr α
3.96 (dd, 14.1, 4.6 Hz, 1H)	3.88 (dd, 14.4, 4.8 Hz, 1H)	-	Tyr β
3.68 (dd, 14.1, 9.2 Hz, 1H)	3.66 (dd, 14.5, 8.4 Hz, 1H)	-	Tyr β

Table S7. Deuterium labelling of exchangeable protons for LRC, tyrosine and xanthurenic acid

LRC, tyrosine (Tyr) and xanthurenic acid (XA; 50 μg/ml) were dissolved in 50% D_2O and 50% CD_3CN with 0.1% formic acid and these materials studied by direct injection using the nanoflow Orbitrap. Under these acidic conditions, the $[LRC+H]^+$ion was found to contain three exchangeable protons while $[tyrosine+H]^+$ had four and $[xanthurenic\ acid+H]^+$ had two rapidly exchanging protons and one slowly exchanging proton (minor).

Ion	Experimental m/z	Calculated m/z	% of pattern
**Unlabeled LRC ions in D_2O:CD_3CN (1:1) with 0.1% formic acid.**			
$[LRC + D]^+$	370.1140	370.1144	20
$[LRC – H + 2D]^+$	371.1203	371.1207	47
$[LRC – 2H + 3D]^+$	372.1260	372.1269	32
**Tyrosine ions in D_2O:CD_3CN (1:1) with 0.1% formic acid.**			
$[Tyr + H]^+$	182.0811	182.0812	29
$[Tyr + D]^+$	183.0870	183.0874	18
$[Tyr – H + 2D]^+$	184.0934	184.0937	11
$[Tyr – 2H + 3D]^+$	185.0998	185.1000	29
$[Tyr – 3H + 4D]^+$	186.1058	186.1063	12
**Xanthurenic acid ions in D_2O:CD_3CN (1:1) with 0.1% formic acid.***			
$[XA + H]^+$	206.0447	206.0448	39
$[XA + D]^+$	207.0506	207.0448	42
$[XA - H + 2D]^+$	208.0565	208.0573	16
$[XA - 2H + 3D]^+$	209.0634	209.0636	<3% -not rapidly exchanging

*XA had two rapidly exchangeable protons under acidic conditions (carboxylic acid and N-H). The molecule also contained one slowly exchangeable proton (less than 5% of the pattern, probably phenol exchange).

SDS-PAGE gels G1 and G2. SDS-PAGE analyses of chromatography of cell lysate from *A. luminosa* light organs (see also Figure 3).

Gel G1: SDS-PAGE of fractions eluted from gel-filtration chromatography. Lane 1, molecular weight markers; lane 2, 15 µl of lysate sample loaded onto column; lanes 3-15, 200 µl of each 1 ml fraction eluted between 7-20 ml (acetone precipitated). Lanes 11 to 15 correspond to the five fractions eluted from the gel-filtration column that contained significant bioluminescent activity (elution volumes 15 to 20 ml), and were pooled for injection onto the ion exchange column.

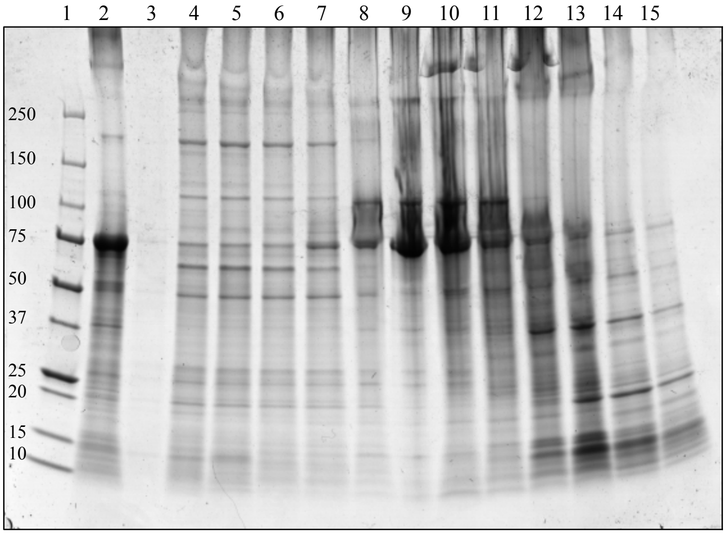

Gel G2: SDS-PAGE of active fractions eluted from ion exchange chromatography. Lane 1, molecular weight markers; lane 2, 40 µl of pooled sample loaded onto column; lanes 3-12, 200 µl of each 1 ml fraction eluted between 10 and 20 ml (acetone precipitated). Lane 7 is the fraction eluted from the ion exchange column with the highest bioluminescent activity.

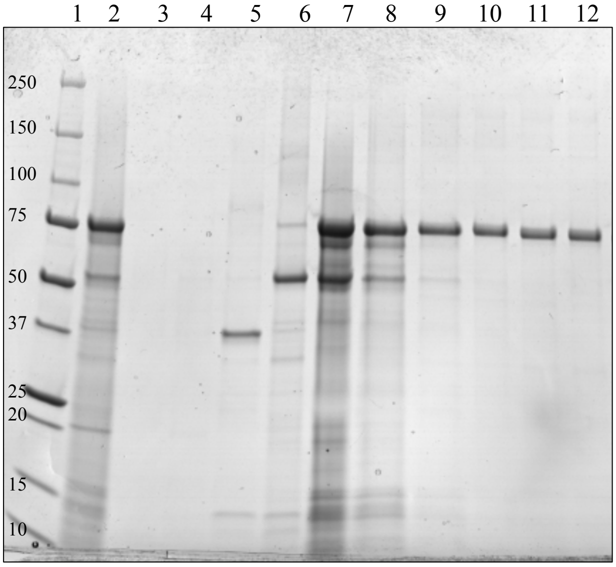

Supplementary Methods

LTQ ion-trap LC-MS

The LC-MS system consisted of a Thermo Electron Corporation (San Jose, CA, USA) Finnigan Surveyor MS pump, Thermo Accela Open Auto sampler (PAL HTC-xt with DLW), Finnigan Surveyor PDA plus detector and a ThermaSphere TS-130 column heater (Phenomenex, Torrance, CA, USA). Aliquots (20 µl) of each prepared sample were separated with a mobile phase consisting of 0.1% formic acid in water (A) and 0.1% formic acid in acetonitrile (B) by reverse phase chromatography (Aqua guard cartridge 4 x 2 mm, 10 µ and Synergi-HydroRP C18, 4 µ, 80 Å, 250 x 2.1 mm, Phenomenex, Torrance, CA, USA) maintained at 30°C with a flow rate of 200 µl/min. HPLC method 1 was applied with both mobile phase A and B containing 0.1% formic acid. The eluent was scanned by photodiode array (200-600 nm) and API-MS (LTQ, 2D linear ion-trap, Thermo-Finnigan, San Jose, CA, USA), with electrospray ionisation (ESI) in the negative or positive ion mode. Data were acquired for parent masses from m/z 145–2000 Daltons with MS^3. Data were processed with the aid of Xcalibur®2.2 (Thermo Electron Corporation).

Orbitrap nanoflow LC-MS

LC: Ultimate 3000 RSLC nano System (DIONEX), Column temperature: 30 °C. Column: C18, DrawSpeed: 200 [nl/s], DrawDelay: 5000 [ms], DispSpeed: 2000 [nl/s], DispenseDelay: 2000 [ms], Pump Flow: 0.500 [µl/min]. Buffer A: 5% milli Q, 95% MeCN, 0.1% FA, Buffer B: MeCN, 0.1% FA. Injection volume (1 µl), Gradient elution: 0 - 3 min (0% B), 3 - 18 min (0 – 15% B), 18 - 20 (15 – 60% B) 20 -22 (60 - 99%B), 22 – 24 (99%B) 24 – 25 (99 – 0% B) and 25- 32 (0% B). **Mass spectrometry:** ThermoScientific LTQ Orbitrap XL **MS:** Scan range: 105-800, Act type: CID, Iso width (m/z): 1, Normalized collision energy: 35, Resolution, 100000. Data type: profile, Analyser FT-MS, Polarity: positive, **MS^2:** Act type: HCD, Iso width (m/z): 1, Normalized collision energy: 45. Activation time: 30 ms (take MS of 4 most intense ion). Data were processed with the aid of Xcalibur®2.2 (Thermo Electron Corporation) and Thermo Scientific Sieve software. Concentrations were calculated from peak areas using the conversion factors in the table below. Conversion factors: peak area / concentration (µm). The concentrations of other compounds were calculated using an adaption factor: $2 * 10^7$.

Compound	RT	Ion m/z	Factor	R^2
3-OH Kynurenine	4.58	225.0867	$2.78 * 10^7$	0.975
UL Tyr	4.59	182.0811	$1.88 * 10^7$	0.971
SIL Tyr	4.66	192.1084	$1.45 * 10^7$	0.945
Guanosine	7.44	284.099	$1.02 * 10^7$	0.882
Phenylalanine	6.71	166.0862	$1.68 * 10^7$	0.783
Tryptophan	10.19	205.0972	$1.37 * 10^7$	0.946
Riboflavin	18.38	377.1457	$2.42 * 10^7$	0.932
xanthurenic acid	14.45	206.0448	$1.63 * 10^7$	0.963
Glut tyr	9.2	311.1229	$1.35 * 10^7$	0.931

Supporting Information references

Branchini, B., Magyar, R., Murtiashaw, M. & Portier, N. (2001) The role of active site residue arginine 218 in firefly luciferase bioluminescence. *Biochemistry* **40**(8): 2410-2418.

Branchini, B., Southworth, T., Murtiashaw, M., Boije, H. & Fleet, S. (2003) A mutagenesis study of the putative luciferin binding site residues of firefly luciferase. *Biochemistry* **42**(35): 10429-10436.

Branchini, B., Southworth, T., Murtiashaw, M., Wilkinson, S., Khattak, N. & Rosenberg, J. (2005) Mutagenesis evidence that the partial reactions of firefly bioluminescence are catalyzed by different conformations of the luciferase C-terminal domain. *Biochemistry* **44**(5): 1385-1393.

Laws, W. R., Ross, J. A., Wyssbrod, H. R., Beechem, J. M., Brand, L. & Sutherland, J. C. (1986) Time-resolved fluorescence and proton NMR studies of tyrosine and tyrosine analogs: correlation of NMR-determined rotamer populations and fluorescence kinetics. *Biochemistry* **25**(3): 599-607.

Viviani, V. R., Hastings, J. W. & Wilson, T. (2002) Two bioluminescent diptera: the North American *Orfelia fultoni* and the Australian *Arachnocampa flava*. Similar niche, different bioluminescence systems. *Photochem Photobiol* **75**(1): 22-27.

Yanshole, V. V., Sherin, P. S., Gritsan, N. P., Snytnikova, O. A., Mamatyuk, V. I., Grilj, J., Vauthey, E., Sagdeev, R. Z. & Tsentalovich, Y. P. (2010) Photoinduced tautomeric transformations of xanthurenic acid. *Phys Chem Chem Phys* **12**(32): 9502-9515.
